# Binding mechanism and biological effects of flavone DYRK1A inhibitors for the design of new antidiabetics

**DOI:** 10.1038/s41598-023-44810-3

**Published:** 2023-10-23

**Authors:** Katarzyna Pustelny, Przemyslaw Grygier, Agata Barzowska, Barbara Pucelik, Alex Matsuda, Krzysztof Mrowiec, Emilia Slugocka, Grzegorz M. Popowicz, Grzegorz Dubin, Anna Czarna

**Affiliations:** 1 Malopolska Centre of Biotechnology, Jagiellonian University, Gronostajowa 7A, 30-387 Krakow, Poland; 2 Doctoral School of Exact and Natural Sciences, Jagiellonian University, Krakow, Poland; 3 Doctoral School of Medical and Health Sciences, Jagiellonian University Medical College, Krakow, Poland; 4 Institute of Structural Biology, Helmholtz Zentrum Munchen, Neuherberg, Germany

**Keywords:** Biochemistry, Drug discovery, Structural biology, Diseases, Molecular medicine

## Abstract

The selective inhibition of kinases from the diabetic kinome is known to promote the regeneration of beta cells and provide an opportunity for the curative treatment of diabetes. The effect can be achieved by carefully tailoring the selectivity of inhibitor toward a particular kinase, especially DYRK1A, previously associated with Down syndrome and Alzheimer's disease. Recently DYRK1A inhibition has been shown to promote both insulin secretion and beta cells proliferation. Here, we show that commonly available flavones are effective inhibitors of DYRK1A. The observed biochemical activity of flavone compounds is confirmed by crystal structures solved at 2.06 Å and 2.32 Å resolution, deciphering the way inhibitors bind in the ATP-binding pocket of the kinase, which is driven by the arrangement of hydroxyl moieties. We also demonstrate antidiabetic properties of these biomolecules and prove that they could be further improved by therapy combined with TGF-β inhibitors. Our data will allow future structure-based optimization of the presented scaffolds toward potent, bioavailable and selective anti-diabetic drugs.

## Introduction

Diabetes mellitus (DM) is a metabolic disorder characterized by elevated blood glucose levels, with insulin resistance and pancreatic beta cells dysfunction, which are two main components of disease pathophysiology^1^. With the observed increase in the number of cases, DM is rising to the epidemic level^2^. Since uncontrolled diabetes can lead to serious health complications and, if left untreated, death, it becomes a major challenge in the management of global public health. While there is currently no cure, recent investigation on beta cells regeneration offers hope for a genuinely curative therapy for diabetes in the near future^3,4^.

Flavones are a class of biologically active secondary plant metabolites that are ubiquitous in fruits, vegetables, nuts, cocoa, tea, cereal seeds, and herbs^5^. In addition, flavones had brought scientific attention to their positive health effects on metabolic disorders, cardiovascular diseases, cancer, obesity, diabetes, and neurological disorders, e. g. Alzheimer’s disease (AD)^6,7^. Yet, limited solubility and bioavailability coupled with rapid degradation in vivo reduce their therapeutic potential^8^. Limited specificity of flavones and the plethora of their targets cause difficulties in evaluation of their mode of action on cellular level^9^. These problems can be overcome by medicinal chemistry tuning of flavones properties and optimal formulations making them an attractive starting point for further drug discovery^10,11^.

Prior research suggests that flavones exert their cellular effects through direct interactions with specific protein targets, central to intracellular signaling cascades^12,13^. In particular, flavones have been demonstrated to interact with various components of kinase signaling cascades, like phosphoinositide 3-kinase (PI3K)^14,15^, protein kinase C (PKC)^16^ and mitogen-activated protein kinase (MAPKs)^17^ or others^18,19^. Additionally, DYRK1A, was identified as a potential new drug target for AD treatment through the analysis of quercetin's pharmacological targets in AD patients^20^. The flat structure of flavones constitutes the ideal scaffold to target the ATP-binding pocket of kinases.

The diabetic kinome is composed of protein kinases known to play a key role in modulating cell processes engaged in diabetes progression^21,22^. Small molecules that target kinase proteins have been shown to be successful in animal models of insulin resistance and diabetes, primarily by re-establishing insulin homeostasis^23,24^. More recently, the modulation of kinase activity involved in regulation of beta cells proliferation and preservation of cell mass has become an attractive approach for intervention in diabetes^25,26^. In 2015, Wang and colleagues^27^, for the first time described a role for Dual Specificity Tyrosine Phosphorylation-Regulated Kinase 1 A (DYRK1A) in human beta cells proliferation and indicated the DYRK1A-NFAT pathway as a control point. Since then, multiple laboratories have reported small molecule DYRK1A inhibitors as putative human beta cells regenerative compounds, both in vitro and in vivo^28^. The DYRK1A inhibitors described so far belong to small chemical molecules selected from both drug discovery programs and natural sources. Of these, harmine, the natural product, and its analogues (β-carbolines) are the most extensively invastigated and remain among the most potent and oral available DYRK1A inhibitors.

Here, knowing that flavones improve cell proliferation and preserve beta cells mass^29–31^, we decided to verify whether DYRK1A kinase is a molecular target responsible for the observed phenotype. We document strong inhibition of DYRK1A by flavones in biochemical assays. Our X-ray structures confirms flavones interaction with DYRK1A kinase. Cellular and pancreatic islets organoid experiments show increased beta cells proliferation and insulin productions in response to flavone treatment. A synergistic effect was observed during the combined therapy of flavones with TGF-β inhibitors, but not GLP-1R agonists.

## Material and methods

### Cell culture

HEK293T cell line was obtained from the European Collection of Cell Culture. Cells were cultured in minimal DMEM medium (Invitrogen) supplemented with 10% fetal bovine serum (Lonza). Cells were maintained at 37 °C in a humidified atmosphere containing 5% CO_2_.

Rat insulinoma (INS-1E) cells were kindly provided by Prof. P. Maechler and were cultured as previously described^32^. INS-1E were grown in DMEM (Pan Biotech) with the addition of 10% fetal bovine serum (Pan Biotech) and supplemented by antibiotics (100 IU·mL^−1^ penicillin and 100 mg·mL^−1^ streptomycin) and 55 µM β-Mercaptoethanol (Pan Biotech, Aidenbach, Germany). Mouse insulinoma (MIN6) cells were grown in DMEM (Pan Biotech) with the addition of 15% fetal bovine serum (Pan Biotech) and supplemented by antibiotics (100 IU·mL^−1^ penicillin and 100 mg·mL^−1^ streptomycin) and 55 µM β-Mercaptoethanol (Pan Biotech). The cells were cultured in incubators maintained at 37 °C with 5% CO_2_ under fully humidified conditions. All experiments were performed on cells in the logarithmic phase of growth. Media were replaced every 2 days, and cells were subcultured using 0.25% trypsin–EDTA (Gibco).

### Plasmid construction

The DNA encoding kinase domain of DYRK1A (126–490) with N-FLAG (MDYKDDDDK), and NFATc1, CyclinD1, both with N-HA (MYPYDVPDYS) tags were synthesized by Genscript and cloned into pcDNA3.1 for eukaryotic cell overexpression. Fluorescent protein fusions were prepared by appending genes encoding mCherry and eGFP using restriction-free cloning^33^.

For bacterial expression, the fragment of gene encoding kinase domain of DYRK1A (126–490) was PCR amplified from pcDNA3.1_DYRK1A plasmid and subcloned into pET24a, using restriction-free method^33^. The kinase domain of DYRK1A (126–490) was expressed together with a non-cleavable C-terminal His-tag.

### Protein expression and purification

DYRK1A was expressed in *E. coli* LOBSTR strain (Kerafast) in LB medium supplemented with kanamycin (50 µg/mL) at 17 °C for 16 h as described previously^34^. Briefly, the pellet was resuspended in cold lysis buffer (20 mM HEPES pH 7.5, 300 mM NaCl, 5% glycerol, 15 mM imidazole and 5 mM β-Mercaptoethanol with EDTA-free Protease Inhibitor Cocktail (Roche)) and the cells were disintegrated by sonication. Clarified lysate was passed through HisPur™ Cobalt resin (ThermoFisher Scientific), and the protein of interest was eluted with increasing imidazole concentration (50–300 mM). The fraction corresponding to DYRK1A was pulled and dialyzed against 20 mM HEPES pH 7.5 containing 50 mM NaCl and 5 mM β-Mercaptoethanol. Next, the protein was purified by an ion-exchange chromatography (HiTrap Q FF column (Cytiva)) and a size exclusion chromatography (HiLoad 16/600 Superdex 75 pg column (Cytiva)). Purified DYRK1A (126–490) kinase domain in a buffer 20 mM HEPES pH 7.5 containing 150 mM NaCl and 5 mM β-Mercaptoethanol was flash-frozen in liquid nitrogen and stored at − 80 °C for further analysis.

### Cook—activity assay

The inhibitory potency (IC_50_) of all compounds was determined in Cook activity assay^35,36^ in which ADP production was linked to pyruvate kinase and lactate dehydrogenase dependent NADH oxidation. The assay was carried out as described previously^37^. The 75 µl of assay mixture (100 mM MOPS pH 6.8, 100 mM KCl, 10 mM MgCl_2_, 1 mM phosphoenolpyruvate, 1 mM peptide substrate DYRKtide (RRRFRPASPLRGPPK, Caslo ApS), 1 mM β-Mercaptoethanol, 15 U/ml lactate dehydrogenase with 10 U/ml pyruvate kinase and 10.7 mM NADH) was mixed with 10 µl of 2.5 µM kinase and 5 µl of a compound in DMSO (20 nM to 200 µM) and incubated for 10 min at room temperature. Then the reaction was started by simultaneous addition of 10 µl of 1280 µM ATP. The NADH conversion velocity was measured at 340 nm for 300 s at room temperature. Control reactions in the absence of peptide substrate were used to detect basal ATPase activity. All measurements were done in triplicate and IC_50_ was determined using GraphPad Prism software.

### CyclinD1 phosphorylation profile

Based on the procedure detailed in Grygier et al.^34^, the experiment was conducted as follows: HEK293T cells were seeded in 24-well plates at the density of 2 × 10^5^ cells/well. After 24 h the cells were co-transfected with plasmids encoding HA-CyclinD1 (0.5 µg) with Flag-DYRK1A or empty vector (0.2 µg) using PEI Prime (Sigma-Aldrich) transfection reagent. 48 h later cells were treated with compounds (10 μM) or DMSO for 6 h and lysed in RIPA buffer supplemented with protease (Sigma-Aldrich) and phosphatase (Calbiochem) inhibitors. Total cell proteins (10 µg, quantified by the BCA method) were separated by 12% SDS-PAGE and transferred to the PVDF membrane (ThermoScientific). Proteins were analysed by Western Blot using anti-FLAG monoclonal antibody (Sigma-Aldrich; F3165) for DYRK1A detection, anti-HA monoclonal antibody (Cell Signaling; C29F4) for CyclinD1 and anti-phoshoCyclinD1 (Thr286) (Cell Signaling; D29B3) for phosphoCyclinD1 and relevant HRP-conjugated secondary antibodies.

### NFAT activity luciferase assay

HEK293T NFAT cells^36^, constitutively expressing DYRK1A, NFAT, and *Renilla* luciferase under the control of an NFAT-RE promoter, were seeded in 96-well white plates (Cellstar, Greiner) at the density of 1 × 10^4^ cell/well. 24 h later cells were pre-treated for 3 h with the indicated concentration of inhibitor, the medium was changed, and cells were stimulated with 1 × Cell Stimulation Cocktail (phorbol 12-myristate 13-acetate (PMA) and ionomycin (IM)) (Thermo Fisher Scientific) for 5 h. Next, the cells were lysed with 1 × Passive Lysis Buffer (Promega) and luciferase activity with *Renilla*-Glo Luciferase Assay System (Promega) was measured. All experiments were done in triplicates.

### NFAT translocation assay

Based on the procedure detailed in Grygier et al.^34^, the experiment was conducted as follows: HEK293T cells were grown on μ-Slide 8 well (IBIDI) to 50–70% confluency. The plasmids expressing the desired proteins, mCherry-DYRK1A and eGFP-NFATc1, were transiently co-transfected with PEI Prime (Sigma-Aldrich). 24 h later cells were pre-treated with compounds (10 μM) or DMSO for 3 h and then stimulated with IM (5 μM) (Thermo Fisher Scientific) for 1 h. Cells were washed with 1 ml PBS and the nuclei were stained with Hoechst 33258 (ThermoScientific) for 10 min at room temperature and fixed with 4% paraformaldehyde in phosphate-buffered saline (PBS) for 10 min at room temperature. Images were collected with Zeiss Axio Observer 3 fluorescence microscope with 40 × objective and analysed in ZEN Blue edition software.

### Protein crystallization, data collection and structure determination

For crystallization, DYRK1A was concentrated to 12–15 mg/ml and the protein was incubated overnight with 5 molar excess of compound at 4 °C. The preparation was mixed 1:1 (v/v) with the crystallization solutions. Crystallization experiments were carried out at 20 °C. Crystals appeared within 1–3 days at room temperature.

DYRK1A/gossypin (PDB: 8C3R) and DYRK1A/rutin (PDB: 8C3Q) complexes were obtained in 0.1 M Bis–Tris pH 5.5, containing 0.2 M ammonium acetate and 25% PEG3350.

Crystals were cryoprotected with mother liquor containing 25% glycerol and flash frozen in liquid nitrogen. Measurements were carried out at BESSY (Berlin, Germany), beamline 14.1, Helmholtz-Zentrum Berlin and DESY (Hamburg, Germany), beamline P11, a member of the Helmholtz Association HGF.

The diffraction data was indexed and integrated in XDS^38^. Data was scaled in AIMLESS^39^ from CCP4 software package^40^. Following steps were performed in Phenix^41^. Both DYRK1A structures were solved by molecular replacement using PHASER^42^ and 6EIS^36^ as search models. Models were refined by interchanging cycles of automated refinement using phenix.refine^43^ and manual building in Coot^44^. Restraints for the inhibitors were created in GradeServer^45^. Data collection and refinement statistics are summarized in Supplementary Table S1.

### Molecular modelling

All simulations, analyses and visualisations were conducted with Maestro software (Schrödinger LLC, New York, NY, 2022-3 Release). Quercetin, herbacetin and baicalein chemical structures underwent energy minimization with LigPrep script with the OPLS4 force field, and the most viable conformations were used in docking studies with Induced Fit Docking script^46^. Molecular modelling was performed with the OPLS4 force field. DYRK1A/rutin (PDB: 8C3Q) crystal structure underwent refinement with Protein Preparation Wizard, with Epik and PROPKA scripts applied to ligand and protein protonation states generation^47^. Prepared in such way, DYRK1A/rutin (PDB: 8C3Q) crystal structure was used as a protein model for docking studies. The following H-bond constraints have been applied in the Induced Fit Docking process: Lys188, Leu241, Phe308. The poses have been selected based on the g-score value and visual analysis. To further investigate interactions and ligand stability in the binding pocket of DYRK1A the molecular dynamics (MD) simulations were conducted with Desmond MD System (D. E. Shaw Research, New York, NY, 2022, Maestro-Desmond Interoperability Tools, Schrödinger, New York, NY, 2022-3 Release)^48^ with prepared protein–ligand complexes, as described above. Systems were solvated in 10 Å cubic box, TIP3P water model in 0.15 mM KCl and neutralized by the addition of 11 Cl^−^ ions, with OPLS4 force field. Atom types and partial charges of ligands from complexes after system preparation are placed in Supplementary Fig. S7. The MD simulations were run in NPT ensemble with default relaxation protocol (Nosé–Hoover chain thermostat method with 1.0 ps relaxation time; p = 1.01325 bar, and Martyna–Tobias–Klein barostat method, 2.0 ps relaxation time, isotropic coupling style, T = 300 K). RESPA integrator with 2, 2, and 6 fs timesteps were used for bonded, near and far, respectively. For short-range Coulombic cutoff, the default value of 9 Å radius was applied. The time of simulation was 100 ns. Interactions lasting more than 30% of the simulation time were documented. Simulation interaction analysis diagrams of RMSD, L-RMSF, P-RMSF have been analysed and placed in Supplementary Fig. S6.

### LogP determination

The partition coefficient was determined by the shake-flask method as described previously^49^. Because the solvents partially dissolve in one another, a small amount of compound was dissolved in 5 mL PBS-saturated n-octanol. The sample was sonicated until all the amount of compound was dissolved. In the next step, 5 mL of PBS saturated with n-octanol was added and the experiment proceeded as before. The resulting mixture was vortexed for 15 min. The sample was centrifuged for 2 min at 3700 rpm to obtain an accurate phase separation. Next, 0.02 mL of each phase was taken and diluted in 3.98 mL DMSO. The next steps included a 5 min sonication and measurement of fluorescence spectra of the obtained solutions. The calibration curve was prepared to determine the concentration of the tested compounds in individual solutions. It was made using the fluorescence intensity of the compounds in series dilution solutions in DMSO with a 0.5% PBS buffer or n-octanol in the concentration range of 1–100 nM.

### Cytotoxicity and cell survival assay

The MTT (3-(4,5-dimethylthiazol-2-yl)-2,5-diphenyl tetrazolium bromide) (Invitrogen) assay was used to quantify cell survival and inhibitor-mediated cytotoxicity as described previously^37^. After cell attachment, compounds in the growth medium at concentrations from 0 to 100 μM were added to the cell cultures. In other experiments the most effective concentration of investigated compounds and 2.5 μM solution of TGF-β inhibitor (LY364947) or 5 nM of GLP-1R agonist (GLP-1) was added to the cell cultures. The treated cultures were incubated for 24 h. Next, the compounds’ solutions were removed, cells were washed in PBS, and a fresh culture medium with FBS and antibiotics was added to each well. MTT dissolved in PBS (Pan Biotech) at content 10% of final solution were added to each well, and the microplates were further incubated for 3 h. The medium was then discarded, and 100 μL of a mixture of DMSO/methanol (1:1) was added to the cultures and mixed thoroughly to dissolve the dark blue crystals of formazan. Formazan quantification was performed using an Infinite M200 Reader (Tecan) automatic microplate reader by absorbance measurements at 565 nm.

### Glucose-stimulated insulin secretion (GSIS)

Glucose-Stimulated Insulin Secretion assay was carried out as described previously^37^. Briefly, cells were cultured in a low glucose medium (5.5 mM) for 24 h. After this medium was changed to lower glucose medium (2 mM) for 2 h and then for high glucose medium (25 mM) for 30 min, media were harvested and/or cells were used for further analysis.

### Staining for insulin and Ki-67

The presence of insulin and Ki-67 was assessed in MIN6 pancreatic cell line as described previously^37^. Before imaging, cells were seeded onto slides at a density of 1 × 10^5^ cells and maintained at 37 °C in 95% atmospheric air and 5% CO_2_ in a humidified atmosphere for 24 h. After washing with fresh medium, cells were incubated with the most effective compound solution for 24 h. After this time, the solution was replaced with a culture medium. The cells were fixed. Then after washing and permeabilization, they were incubated with anti-Insulin and anti-Ki-67 monoclonal antibodies (Invitrogen, 2488619; 2172694) diluted in PBS. Next, the cells were incubated for 10 min with Hoechst33342 (Sigma-Aldrich). After incubation, at 37 °C, in the dark, the cells were washed twice with HBSS (Gibco), the slide was transferred to a microscope table, and the cells were visualized under a Zeiss LSM 880 confocal microscope (Carl Zeiss, Jena, Germany) with a 40 × immersion objective. Images were analyzed by Zeiss ZEN Software.

### Flow cytometry analysis

The presence of insulin was assessed in MIN6 and INS-1E^32^ pancreatic cell lines. Before analysis, cells were seeded onto wells at a density of 1 × 10^4^ cells and maintained at 37 °C in 95% atmospheric air and 5% CO_2_ in a humidified atmosphere for 24 h. After washing with fresh medium, cells were incubated with a compound solution for 24 h. After this time, the solution was replaced with a culture medium. The medium was changed every 2 days. At selected days (1, 2, 3, 12) the cells were detached from the plate using trypsin. The cells were fixed. Then after washing and permeabilization, they were incubated with specific antibodies (Invitrogen, 2488619) diluted in PBS. Insulin content in cells was measured with Guava® easyCyte™ flow cytometer equipped with a 488 nm laser. The obtained data were analyzed using InCyte software (MerckMillipore, Burlington, MA, USA).

### Differentiation of hiPSC into pancreatic islets cells

Differentiation of hiPSC into iPSC-derived β-cell islets was carried out following the protocol established by Pellegrini et al.^50^. A day before differentiation, 1 × 10^4^ cells were seeded on coated with Geltrex (Gibco) 12 wells plate in Essential 8 medium (Gibco) with Y-27632 10 µM (Abcam). The appropriate culture media and required components based on the protocol of Pellegrini with modifications, as published in Barzowska et al.^37^.

### ELISA assay

The Insulin ELISA(Invitrogen Insulin Mouse ELISA Kit, Invitrogen Insulin Human ELISA Kit) was used to quantify insulin levels in control and treated cells and organoids. Cells and organoids were homogenized in lysis buffer. Obtained cell lysates and medium samples (after GSIS) were analysed for total protein level and subsequently processed following the manufacturer’s protocol.

### Statistical analysis

Data are presented as mean ± standard deviation (SD) or standard error of the mean (SEM of N independent experiments (at least N = 3)). Statistical significance of differences was assessed using GraphPad Prism 5.0 (GraphPad Software, San Diego, CA, USA). The differences between groups were compared using a two-way ANOVA, and p < *0.05, **0.01, ***0.001 were considered significant.

## Results and discussion

### Identification of flavones as a novel inhibitors of DYRK1A

**Figure 1:**
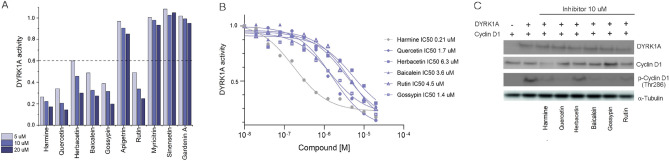
Selected flavones are potent inhibitors of DYRK1A. (**A**) The ability of selected compounds to inhibit DYRK1A activity was determined in the Cook assay. The 5 μM, 10 μM and 20 μM of the compound were incubated with 0.25 mM DYRK1A for 15 min in the reaction mixture and the reaction was initiated by the addition of ATP. The DYRK1A activity after incubation with DMSO was taken as a reference (100%) for the calculation of the residual activity after incubation with compounds. Harmine was used as a positive control. (**B**) IC_50_ values were determined in the same assay condition for compounds’ concentrations of 20 nM—200 μM. (**C**) Selected natural compounds inhibited DYRK1A-dependent CyclinD1 phosphorylation in mammalian cells. HEK293T cells were transfected with plasmids expressing HA-CylcinD1 and Flag-DYRK1A or empty vector. 48 h after transfection the cells were incubated for 6 h with a vehicle or the indicated inhibitor (10 μM). Total cell extracts were prepared and subjected to Western blotting with anti-FLAG monoclonal antibody for DYRK1A, anti-HA monoclonal antibody for CyclinD1 and anti-phoshoCyclinD1 (Thr286). α-Tubulin protein was used as a control. Western blotting was performed twice, and representative data are presented.

The DYRK1A kinase has been implicated in various human diseases, mainly in diabetes mellitus and neurological disorders^77^. Both diseases are primarily caused by lifestyles, aging, and genetic alterations and share several mechanisms on a molecular level. Flavones are naturally occurring compounds whose dietary intake is inversely related to the occurrence of DM and AD^78^. We decided to check the ability of flavones to modulate DYRK1A activity. We tested 9 natural flavones: aglycones (quercetin, herbacetin, baicalein), glycosides (gossypin, apigetrin, rutin, myricitrin) and methylated derivatives (sinensetin, gardeninA) selected based on a literature search. We selected compounds that showed activity both in diabetes and neurological context (Table 1). Initially, we evaluated the compounds’ ability to inhibit DYRK1A-catalyzed phosphorylation of DYRKtide peptide in an ATP regeneration assay, were ADP production was linked to puryvate kinase (PK) and lactate dehydrogenase (LDH) dependent NADH oxidation^35^. To exclude impact of analyzed compounds on PK and LDH enzymes the control reaction were carried out (Supplementary Fig. S5), and demonstrated that the observed inhibition was specific for DYRK1A. In the assay, the truncated DYRK1A protein restricted to the kinase domain (126–490) was used. The residual activity of kinase was determined for three consecutive concentrations of the compound (5 μM, 10 μM, and 20 μM). Among tested compounds, no influence on DYRK1A activity was observed in the case of methylated derivatives (sinensetin and gardenin A) and also two glycosyloxyflavones, with sugar group substituted at position R7 (apigetrin) and R3 (myricitrin) (Fig. 1A). The five compounds, two glycosides with sugar moiety at R3 (rutin) and R8 (gossypin) and three aglycones (quercetin, baicalein and herbacetin), decreased DYRK1A activity dose-dependently. The residual DYRK1A activity dropped below 60% for the lowest analysed concentration of the compounds. For those compounds, IC_50_ values were determined in concentrations ranging from 20 nM to 200 μM and with fixed concentrations of DYRK1A (0.25 mM), ATP (128 µM), and substrate peptide (0.25 mM). The ATP concentration was chosen according to the experimentally determined K_M_ of 118 μM for DYRK1A. All tested compounds inhibited the activity of DYRK1A in an ATP-competitive manner with the IC_50_ below 10 µM. The most active compounds were quercetin and gossypin which inhibited the phosphorylation of the substrate peptide with the IC_50_ values of 1.7 and 1.4 µM, respectively (Fig. 1B).

**Table 1:** Structures of selected flavones.

Compound		IUPAC name									References
			R3	R5	R6	R7	R8	R3’	R4’	R5’	
Aglycones	Baicalein	5,6,7-trihydroxy-2-phenylchromen-4-one	–H	–OH	–OH	–OH	–H	–H	–H	–H	^51–54^
	Herbacetin	3,5,7,8-tetrahydroxy-2-(4-hydroxyphenyl)chromen-4-one	–OH	–OH	–H	–OH	–OH	–H	–OH	–H	^55–57^
	Quercetin	2-(3,4-dihydroxyphenyl)-3,5,7-trihydroxychromen-4-one	–OH	–OH	–H	–OH	–OH	–H	–OH	–OH	^58–61^
Methylated derivatives	Gardenin A	5-hydroxy-6,7,8-trimethoxy-2-(3,4,5-trimethoxyphenyl)chromen-4-one	–H	–OH	–OCH_3_	–OCH_3_	–OCH_3_	–OCH_3_	–OCH_3_	–OCH_3_	^62^
	Sinensetin	2-(3,4-dimethoxyphenyl)-5,6,7-trimethoxychromen-4-one	–H	–OCH_3_	–OCH_3_	–OCH_3_	–H	–H	–OCH_3_	–OCH_3_	^63,64^
Glycosides	Apigetrin	5-hydroxy-2-(4-hydroxyphenyl)-7[(2*S*,3*R*,4*S*,5*S*,6*R*)3,4,5- trihydroxy-6-(hydroxymethyl)oxan-2-yl]oxychromen-4-one	–H	–OH	–H	–O–Glu	–H	–H	–OH	–H	^65,66^
	Gossypin	2-(3,4-dihydroxyphenyl)-3,5,7-trihydroxy-8-[(2*S*,3*R*,4*S*,5*S*,6*R*)-3,4,5-trihydroxy-6-(hydroxymethyl)oxan-2-yl]oxychromen-4-one	–OH	–OH	–H	–OH	–O–Glu	–OH	–OH	–H	^67–69^
	Myricitrin	5,7-dihydroxy-3-[(2*S*,3*R*,4*R*,5*R*,6*S*)-3,4,5-trihydroxy-6-methyloxan-2-yl]oxy-2-(3,4,5-trihydroxyphenyl)chromen-4-one	–O–Rha	–OH	–H	–OH	–H	–OH	–OH	–OH	^70–72^
	Rutin	2-(3,4-dihydroxyphenyl)-5,7-dihydroxy-3-[(2*S*,3*R*,4*S*,5*S*,6*R*)-3,4,5-trihydroxy-6-[[(2*R*,3*R*,4*R*,5*R*,6*S*)-3,4,5-trihydroxy-6-methyloxan-2-yl]oxymethyl]oxan-2-yl]oxychromen-4-one	–O–Rha–Glu	–OH	–H	–OH	–H	–H	–OH	–OH	^73–76^

The five compounds with in vitro inhibitory activity were subsequently tested in a cellular assay to determine if they possessed the ability to inhibit DYRK1A-dependent CyclinD1 phosphorylation. To exclude endogenous kinases inhibition the activity of the compounds was determined in transiently transfected cells. The expression vectors encoding HA-tagged CyclinD1 and Flag-tagged DYRK1A (full length) were co-transfected into HEK293T cells and phosphorylation profile of CyclinD1 (p-CyclinD1) at Thr289 was inspected by Western Blot (Fig. 1C). Thr289 was selected as a known phosphorylation site of DYRK1A kinase and independent to CyclinD-dependent kinases^79^. The assay was insensitive to endogenous kinases, while phosphorylation of CyclinD1 at Thr289 was induced only by the overexpression of DYRK1A. Treatment with 10 μM of rutin, gossypin, quercetin and baicalein significantly reduced CyclinD1 phosphorylation. Moreover, CyclinD1 phosphorylation was at a level comparable to that detected for the treatment with harmine, a known potent inhibitor of DYRK1A. Moderate inhibition of CyclinD1 phosphorylation was observed after treatment with herbacetin.

**Figure 2:**
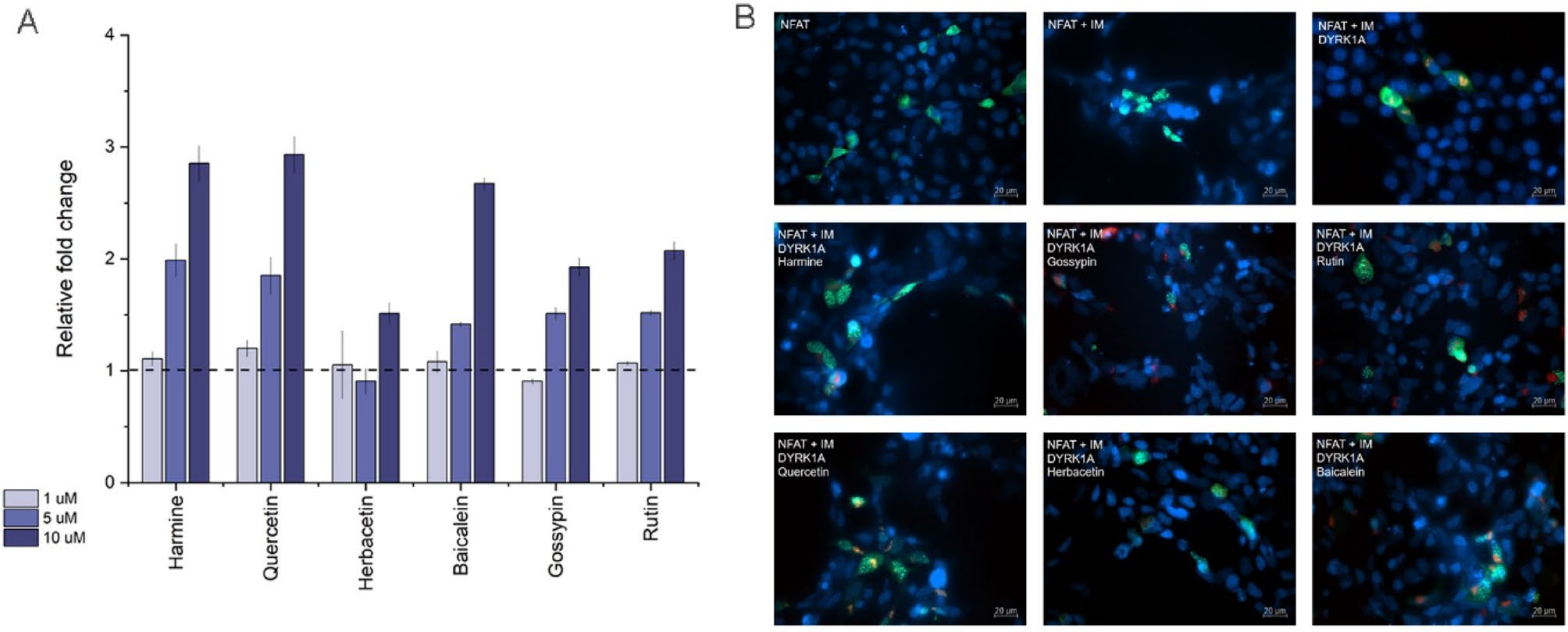
The influence of flavonoids on DYRK1A-related NFAT signaling. (**A**) The effect of natural compounds on NFATc1-mediated transcriptional activity was evaluated in HEK293 NFAT cell line with constitutive expression of DYRK1A, NFAT and *Renilla* luciferase under the control of a NFAT-RE promoter. The cells were pre-treated for 3 h with indicated doses of natural compounds and then stimulated with IM (5 μM) and PMA (10 nM) for 5 h. Next, the cell lysate was harvested, and luciferase activity was measured. Luciferase activity in the sample treated with DMSO was set to 1, and the relative luciferase activities were calculated. Means ± SD were determined from three independent experiments. (**B**) The effect of compounds on calcineurin-NFAT signaling was analysed by visualizing the translocation of the NFATc1 protein. HEK293T cells were transfected with plasmids expressing GFP NFATc1(green) and mCherry DYRK1a (red) or empty vector and then treated for 3 h with indicated compound (10 μM) or DMSO prior to 1 h stimulation with IM (5 μM). The nuclei were stained with Hoechst 33258 (blue). Representative images are presented. Scale bar 20 µm. Images were analyzed by Zeiss Software ZEN Blue version 3.1.

Among others, NFAT transcription factor (Nuclear factor of activated T-cells), the key cell cycle regulator, is one of the primary cellular targets of DYRK1A^80^, either for neurodegenerative processes or beta cells proliferation. DYRK1A turns off the NFAT transcription factor by phosphorylating its nuclear pool and leads its export to the cytosol. Consequently, we decided to study the selected natural compounds for their ability to modulate the DYRK1A regulation of calcineurin/NFAT pathway. First, we used the luciferase NFAT reporter activity test in HEK293T cells with stable expression of DYRK1A, NFAT and *Renilla* luciferase under the control of a NFAT-RE promoter^36^. The cells were titrated with increasing concentrations of compounds ranging from 1 to 10 μM, and the results were compared with the harmine treatment. All compounds except herbacetin dose-dependently activated NFAT response (Fig. 2A). In this test, the most active compounds were quercetin and baicalein, which at a concentration of 10 μM showed an approximate threefold increase in luciferase activity and mimicked harmine treatment. The less active were glycosyloxyflavones compounds (rutin and gossypin), where the 10 μM concentration of the compound induced a twofold increase in *Renilla* luciferase activity. Again, herbacetin was found to be the least potent compound, only effective at the highest concentration tested.

The effect of natural compounds on NFAT signaling was investigated through a complementary assay that monitored the translocation of NFAT-GFP. In untreated cells the overexpressed NFAT-GFP remained mostly in cytosol (Fig. 2B). After cell stimulation with the ionomycin, NFAT-GFP was translocated into the nucleus due to NFAT dephosphorylation by activated calcineurin. Simultaneous overexpression of mCherry-DYRK1A and NFAT-GFP led to its relocation to the cytoplasm even in the presence of stimuli. Both flavones and harmine, at 10 μM concentration, induced nuclear translocation of NFAT-GFP, despite the overexpression of mCherry-DYRK1A. The phenomenon was observed for all the compounds tested and supports our prior conclusion that the concentration of 10 μM is sufficient to observe modulation of DYRK1A- controlled calcineurin/NFAT pathway by tested compounds in the cell model.

### Structural basis of DYRK1A inhibition by flavones

In order to determine at a molecular level the interaction of the analysed compounds with the DYRK1A kinase, we carried out crystallisation attempts. For the determination of the crystal structure, we choose a construct expressing the residues 126–490 of human DYRK1A including the DH box and kinase domain. The crystallization trials allowed us to obtain kinase crystals with glycosylated flavones—gossypin and rutin.

**Figure 3:**
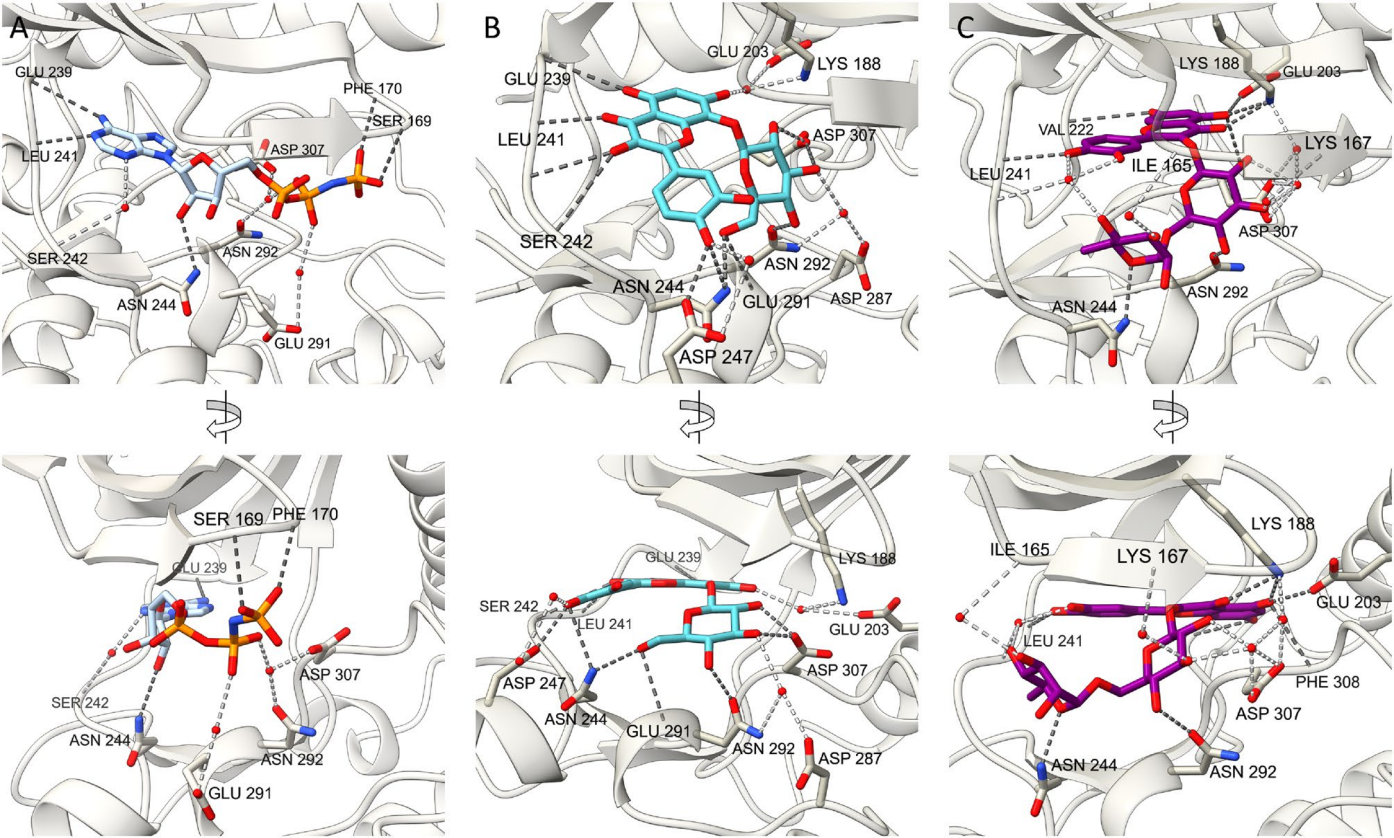
The crystal structures of ATP analogue (AMP-PNP), gossypin and rutin bound to the active site of DYRK1A. The enlargement showing (**A**) ATP analogue (PDB: 7A4O), (**B**) gossypin, (**C**) rutin interaction in the ATP-binding pocket. Direct hydrogen bonds are shown in dark gray dashes and water-mediated hydrogen bonds are shown in light gray. Water molecules are represented as red spheres. Figure was prepared in UCSF ChimeraX 1.6.1 (https://www.cgl.ucsf.edu/chimerax/) and GIMP 2.10.34 (https://www.gimp.org/).

The structures were determined at 2.06 Å and 2.32 Å resolution for gossypin and rutin complexes respectively. The protein-inhibitor complexes crystallized in the P 63 space group with two kinase molecules per asymmetric unit. For both DYRK1A complexes the entire catalytic domain was well ordered, including a long hairpin-like structure for the N-terminal DH box and an active kinase conformation with a fully ordered activation segment, and well-defined electron density of inhibitor in ATP binding pocket (Fig. 3, Supplementary Figs. S1, S2). The DYRK1A-gossypin and DYRK1A-rutin structures superimpose with a root-mean-square deviation (rmsd) of 0.361 Å over 298 Cα atoms. The main differences are seen in hinge region connecting N- and C-lobes and are triggered by distinct mode of inhibitors binding. The binding mode of DYRK1A-gossypin (Fig. 3B) closely resembles the binding of the nonhydrolysable ATP analogue AMP-PNP to DYRK1A (PDB: 7A4O) (Fig. 3A). The hydroxy-chromone moiety of gossypin (ring A and C) interacts principally with DYRK1A hinge region via four H-bonds with main chain atoms of Glu239, Leu241, and side chain of Ser242, which are equivalent to the interaction of DYRK1A hinge region with the adenosine moiety of ATP analogue. In the formed hydrogen bond network, gossypin’s hydroxyl moieties at the R5 and R4 act as the hydrogen bond donors, while the carbonyl group (C4) is the H-bond acceptor. The phenyl moiety of gossypin (ring B), occupies the DYRK1A pocket intended to bind the sugar moiety of ATP. The hydroxy group at the R3’ and R4’ form direct and water-mediated H-bonds with side chains of Asn244 and Asp247. The glycosyl group of gossypin directly interacts with side chains of Asn244, Glu291, Asn292 and Asp307, the residues involved in positioning of α and β phosphate groups of ATP. Contrary to the DYRK1A-gossypin structure, in the DYRK1A-rutin complex (Fig. 3C), the compound is buried deep in the ATP binding pocket with the flavonoid moiety adopting an orientation opposite to that found for gossypin. The phenyl moiety of rutin (ring B) points at DYRK1A hinge region and makes only one direct H-bond between hydroxyl group at R4’ and main chain amide of Leu241. In addition, inhibitor-hinge region interaction is stabilized via water-mediated contacts. The rutin hydroxy-chromone (ring A and C) is located opposite to the DYRK1A hinge region deep inside the ATP pocket, where it docks through H-bond to Val222. Additionally, the hydroxy group at R5 and the carbonyl group (C4) form a direct H-bond with side chain of catalytic Lys188 and side chain of Glu203, a segment known as positive electrostatic area within the kinases structure. The Lys188 targeting, well preserved within the DYRK1A-rutin complex, is a common feature of many DYRK1A inhibitors. However, in the gossypin complex, only weak water-mediated contacts between inhibitor and Lys188 are observed. The pronounced difference between rutin and gossypin is not only the position of glycosylation (C3 of rutin and C8 of gossypin) but also presence of two sugar groups (glucose and rhamnose—rutin) instead of one (glucose—gossypin). In rutin complex the glucose moiety contributes water-mediated contacts with catalytic Lys188 and Asp307 of DFG motive, while rhamnose moiety targets with direct H-bonds the Asn244 and Ile165 from glycine riche loop. Despite the highly hydrophilic nature of both compounds, which is also reflected in the water molecules bound in the part of the ligand exposed to the solvent (Supplementary Fig. S3), the classic hydrophobic interactions at the interior of the ATP pocket are retained. The hydrophobic interaction involves the kinase residues from the N-lobe (Val173 and Phe238) and C-lobe (Met240, Leu294 and Val306). Both compounds interact with the gate keeper residue Phe238 via classic pi–pi interaction.

**Figure 4:**
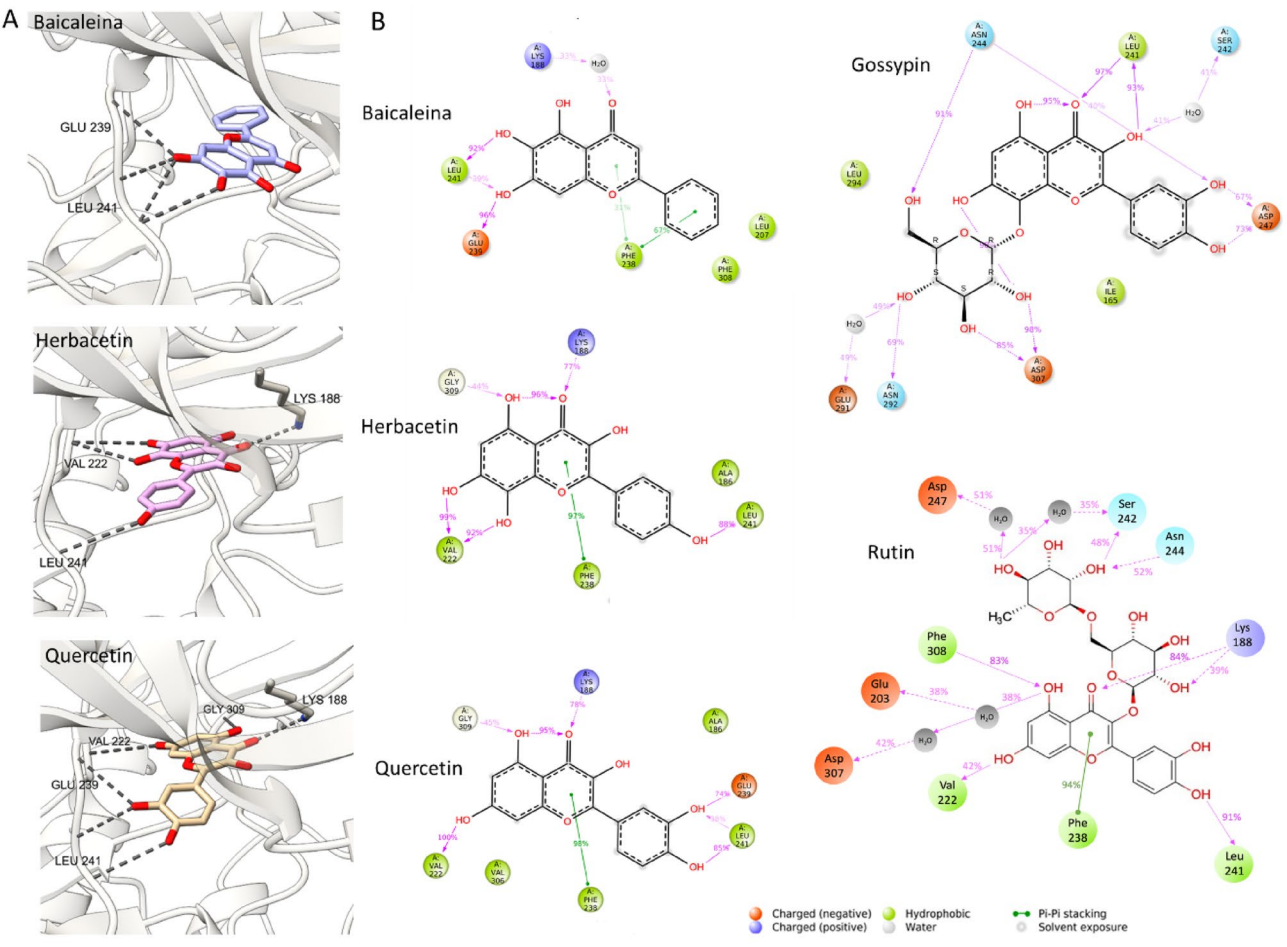
The docking poses of baicalein, herbacetin and gossypin with DYRK1A. (**A**) Inhibitor binding pose in an ATP-binding pocket of DYRK1A modelled with Maestro software. DYRK1A (gray) in cartoon representation with baicalein (blue sticks), herbacetin (pink sticks) and quercetin (yellow sticks) are shown. Hydrogen bonds are shown in gray. (**B**) The schematic representation of detailed ligand atom interactions with the DYRK1A residues derived from molecular dynamics. The interactions that occurs for more than 30% of the simulation time (100 ns) are indicated. Figure was prepared in (A) UCSF ChimeraX 1.6.1 (https://www.cgl.ucsf.edu/chimerax/) and GIMP 2.10.34 (https://www.gimp.org/) (**B**) Maestro-Desmond Interoperability Tools, Schrödinger, Release 2022–3 (https://www.schrodinger.com).

To learn more about the interaction of the other flavones with DYRK1A we run molecular dynamics simulation and analyses with Maestro software. All analysed flavones form lasting interaction (participating in 96%, 88% and 85% of the total simulation time, respectively for baicalein, herbacetin and quercetin), with the Leu241—residue in the hinge region, crucial for the inhibitory activity of the ligand. Quercetin and herbacetin (Fig. 4A,B) arrange their stable position, corresponding with the cocrystal structure of rutin (Fig. 3A) (the glycoside of quercetin) in the way that enables the formation of H-bonds with Leu241 via phenyl moiety (ring B). The hydroxy-chromone moiety (ring A and C) is anchored in hydrophobic region by the H-bonds with Val222, Lys188 and sandwiched by staggered pi-pi stacking with Phe238, the gatekeeper residue. The presence of hydroxy group at R8 in herbacetin reinforce the H-bond interaction with the main chain of Val222, whereas the hydroxy group at R4’ in the phenyl moiety (ring B) of quercetin strengthened the binding with hinge region via H-bond formation with Glu239. Baicalein has a distinct pattern of hydroxyl substitution, that affects the binding pose and interaction formed in the binding pocket. Baicalein is anchored in the hinge region by the two hydroxy groups at R5 and R6 of the hydroxy-chromone moiety (ring A and C) via H-bond with main chain atoms of Glu239 and Leu241 (Fig. 4A,B) which participate in 96% and 92%, respectively, of the time of the simulation. The phenyl moiety (ring B) is sandwich by the pi-pi stacking with Phe238. To verify obtained results we used the same parameters to run the simulation for interaction of gossypin and rutin with DYRK1A and compared them with solved crystal structure. The protein–ligand complexes were correctly reproduced thus validating the procedure used for molecular modelling. The DYRK1A-gossypin forms lasting interaction between hydroxy-chromone (ring A and C) and Leu241 (97% and 93%), phenyl moiety (ring B) and Asp247 (73%), sugar moiety and Asn244 (91%), Asn292 (69%) and Asp307 (98%). In the case of DYRK1A-rutin lasting interaction included phenyl moiety (ring B) and Leu241 (91%), hydroxy-chromone (ring A and C) and Lys188 (84%) and Val222 (99%), while interactions with sugar moiety are weak an only lasted less than 50% of the total simulation time.

The RMSD analysis of molecular dynamic simulation trajectory indicated that all studied protein–ligand complexes are stable and no large conformation changes occurred during the simulation time (Supplementary Fig. S6). The complex reached equilibrium during the 100 ns time of simulation. Rapid fluctuations of ligand and protein RMSD plots at the beginning of the simulation with high variances of deviations reflect release of intermolecular and intramolecular tensions and strains. Ligand RMSD plot for aglycons: baicalein, herbacetin and quercetin demonstrate high stability and low variance of deviation through simulation time. Lack of flexible carbohydrate fragments render the molecules position more stable within the binding pocket. While baicalein and herbacetin are stable through whole simulation, for quercetin the rapid change in RMSD value is noted around the frame at 80 ns. Taking into consideration the L-RMSF for quercetin, it can be explained by the position shift of R5’ located hydroxyl group of the B-ring, forming H-bonds with hinge region residues Leu241, Glu239. L-RMSF plots underlined the distinct binding modes between baicalein and other flavone aglycons. Baicalein B ring is buried within the binding pocket of the DYRK1A, and L-RMSF for indexed atoms is lower (0.2 Å) that for the same placement in herbacetin (1.2 Å) and quercetin (1.2–1.7 Å) which are bounded with hinge region via ring B substituted hydroxyl groups thus exposing the B ring to solvent. Glycosides—rutin and gossypin—manifest higher average RMSD variance than aglycons, explained by the more complex structure and unstable interaction pattern. The available hydroxyl groups can act as H-bond donors and acceptors, and form water mediated hydrogen bonds that contributes to the molecule flexibility. L-RMSF and RMSD of protein-glycosides complexes varies more than aglycons, with the disaccharide rutin demonstrating the highest L-RMSF shifts (around 2.0 Å) and RMSD variances caused by the mobility of the glucose moiety.

The presented crystal structures and molecular modeling analysis revealed that all selected flavones interact with residues Glu239 and Leu241 in the hinge, essential for DYRK1A inhibition. Beyond inhibitor interaction with hinge region (classical for Type I inhibitors), the flavones exhibit two distinct binding patterns: inhibitor oriented toward the kinase P-loop or the front cleft. In the first scenario characteristic for complexes of DYRK1A and rutin, quercetin and herbacetin, inhibitor binds deep in ATP-binding pocket and interact with the catalytic Lys188 and Asp307 of Asp-Phe-Gly motif (DFG). This pattern of interaction is commonly employed in the design of kinase inhibitors^81^, and most investigated inhibitors shared the same mechanism of action targeting both Leu241 and catalytic Lys188. The unexpected biding pattern was observed for gossypin-DYRK1A, where the inhibitor apart from interaction with hinge region and water-madieted interaction with catalytic Lys188, forms unique H-bonds between inhibitor’s catechol moiety and DYRK1A’s Asn244 and Asp247 residues at solvent-exposed region. Interactions with the Asn244 and Asp247 residues of DYRK1A are seldom explored in inhibitor design and only a limited examples could be found such as DANDY’s^82^, AZ-191^83^, KS40008, and GNF4877^84^. Such rare interactions provide interesting starting point for inhibitor specificity optimization.

### Flavones as a new tool to promote beta cells proliferation and insulin secretion

#### Simultaneous treatment with flavones and TGFβ inhibitor or GLP-1R agonist promotes insulinoma cells viability and proliferation

**Figure 5:**
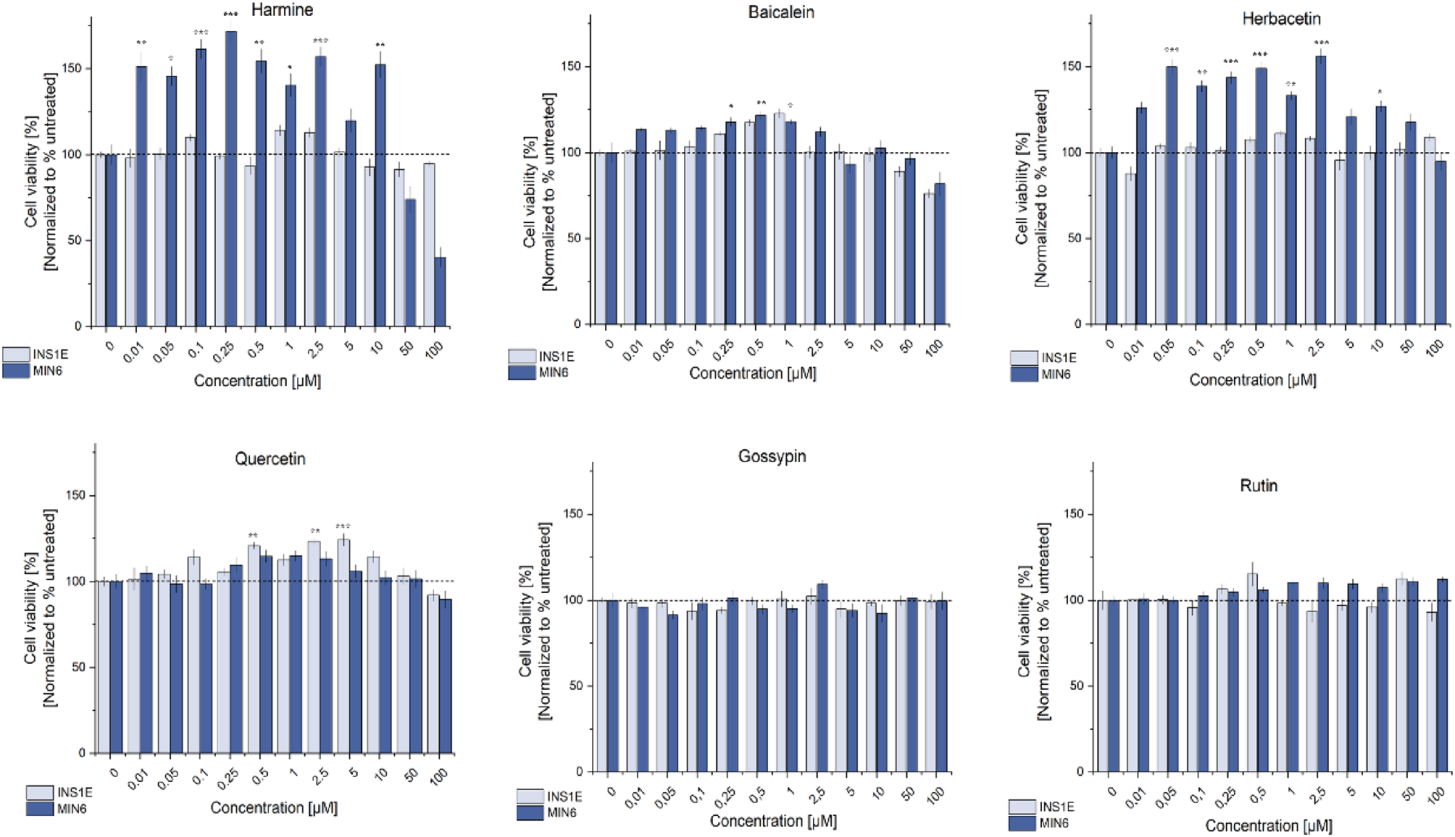
Relative viability of the MIN6 and INS-1E cell lines treated with harmine, baicalein, herbacetin, quercetin, gossypin and rutin. Cell viability, expressed as % of the viability of control cells, was assessed with the MTT assay. Data are expressed as mean ± SEM. The asterisks denote *p*-values < *0.05, **0.01, ***0.001 compared to control.

Regenerative diabetes treatment will most likely require combined therapy, where multiple cellular targets are addressed simultaneously. Wang et al*.* found that the combination of DYRK1A inhibitor with TGFβ inhibitor or GLP-1R agonist can have a synergistic effect^85,86^. Thus, in our study we focused on both separate natural compound treatments and in the combination with TGFβ inhibitor (LY364947) or GLP-1R agonist (GLP-1). For the initial evaluation of compounds, we selected rodent-derived insulinoma cell lines INS-1E (rat) and MIN6 (mouse), which are widely used beta cells surrogates. The influence of natural compounds on the viability of MIN6 and INS-1E cells was examined with a MTT assay. The insulinoma cells were incubated with the five natural compounds (baicalein, herbacetin, quercetin, gossypin, and rutin) or harmine, as the positive control, in a concentration range between 0.01 and 100 μM. For both cell lines the results confirmed that all natural compounds are non-toxic in a used concentration range (Fig. 5). Mild toxicity was observed for harmine at concentrations higher than 50 μM in MIN6 but not in INS-1E. The MTT test also provided information on the effect of compounds on cell viability. Harmine treatment resulted in the robust increased in cell viability in MIN6 cells over a broad range of concentrations (0.01–10 μM). Whereas for INS1-E, the effect was not so significant and involved only a narrow range of concentrations (1–2.5 μM). Identical results were observed for herbacetin. The positive impact on cell viability was also observed for baicalein and quercetin for both cell lines and concentration range 0.1–2.5 μM and 0.25–5 μM, respectively. While treatment with gossypin and rutin did not influence cell viability.

**Figure 6:**
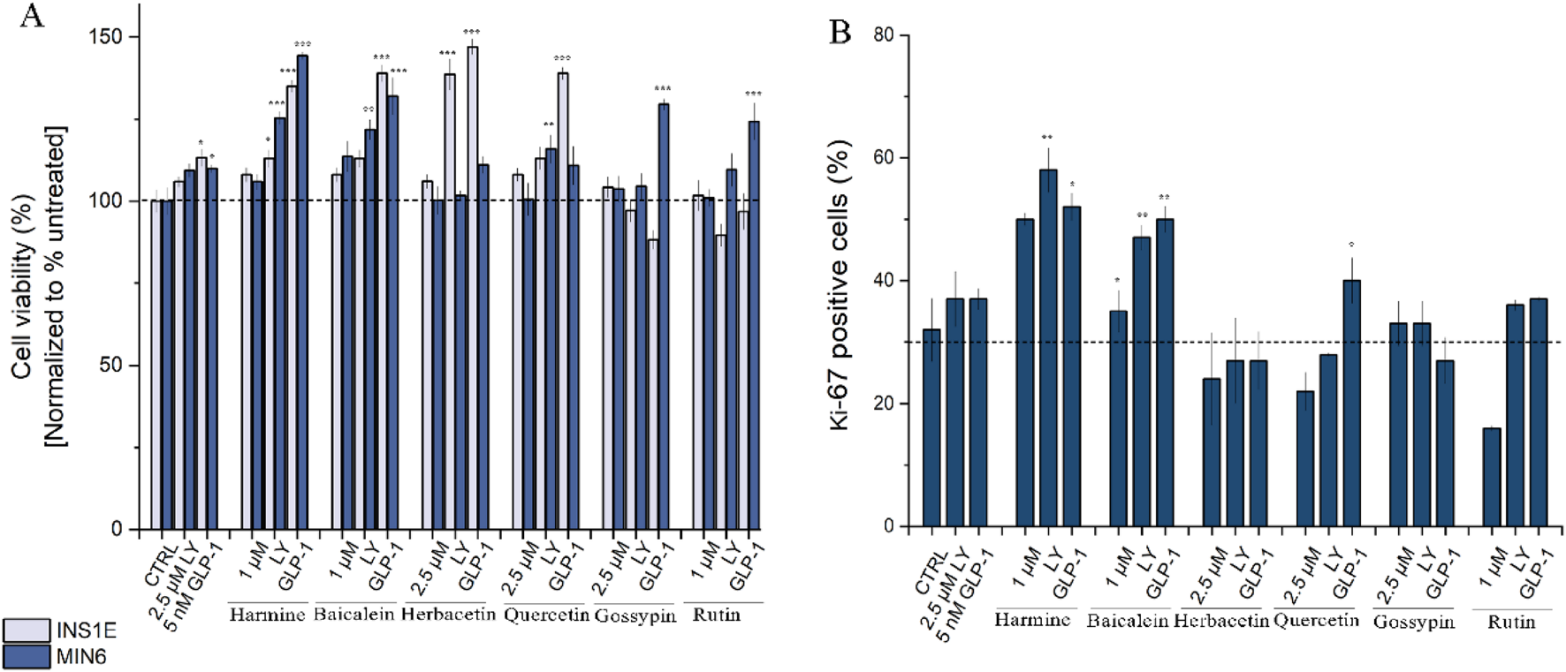
The influence on cell viability and proliferation of combined treatment of insulinoma cell lines with natural compounds and TGFβ inhibitor or GLP-1R agonist. (**A**) The relative viability of the INS1E and MIN6 cell lines treated with LY364947 (2.5 μM) or GLP-1 (5 nM) in combination with harmine (1 μM), baicalein (1 μM), herbacetin (2.5 μM), quercetin (2.5 μM), gossypin (2.5 μM) and rutin (1 μM). (**B**) The analysis of combinatory treatment on MIN6 cells proliferation measured by Ki-67 staining and flow cytometry analysis. All data are expressed as mean ± SEM. The asterisks denote p-values < *0.05, **0.01, ***0.001 compared to the control.

Next, MIN6 and INS-1E cells were treated with the selected concentrations of natural compounds with a combination of LY364947 (2.5 μM)^85^ or GLP-1 (5 nM)^86^ and the MTT assay was performed. Generally, no cytotoxic effect was observed (Fig. 6A), and only treatment with gossypin or rutin in combination with LY364947 or GLP-1 showed a slight cytotoxic effect on INS-1E. Otherwise the simultaneous treatment led to an increase in cell viability for INS1E cells. The GLP-1 in combination with natural compounds (harmine, baicalein, herbacetin and quercetin) increased cell viability by more than 30%, while the combination of compounds with LY364947 had only a significant effect for herbacetin and moderate effects for the remaining compounds. For MIN6 cells the most significant effect was observed for combination of LY364947 and baicalein or quercetin, were the 21% and 16% increase in cell viability over control was observed, respectively. These inhibitors were only slightly less effective than harmine, where 25% increase of cell viability was observed. For the combinatorial treatment with GLP-1R agonist the most potent were baicalein, gossypin and rutin, which showed over 20% increase over control, but did not reach the level observed for harmine (44%).

We then verified if the observed increase in cell viability was linked to enhanced proliferation. The level of MIN6 cell proliferation was estimated by staining with Ki-67, a known indicator of cell proliferation^87^. The data revealed that the control cells population is characterized by 32% of Ki-67-positive cells (Fig. 6B). Interestingly, the treatment with LY364947 and GLP-1 alone induces a slight increase in proliferation rate (up to 5%). Harmine, the positive control, leads to about 20% increase in number of Ki-67-positive cells (50% vs 32% in control cells). The addition of LY364947 and GLP-1 potentiates this effect, while 58% and 55% of Ki-67-positive cells may be observed in a population. Of the natural compounds tested, only baicalein showed a slight increase in the proliferation rate (5%), whereas for others, the rate of cellular proliferation declined slightly. The simultaneous administration of baicalein with LY364947 or GLP-1 significantly enhances cell proliferation (48% and 55%, respectively) also a modest effect was observed for combination of quercetin with GLP-1. No impact on cell proliferation was detected for other condition tested. The Ki-67 based proliferation data were consistent with the MTT results.

#### Combined treatment with flavones and TGFβ inhibitor/GLP-1R agonist improve insulin expression and secretion in insulinoma cell lines

**Figure 7:**
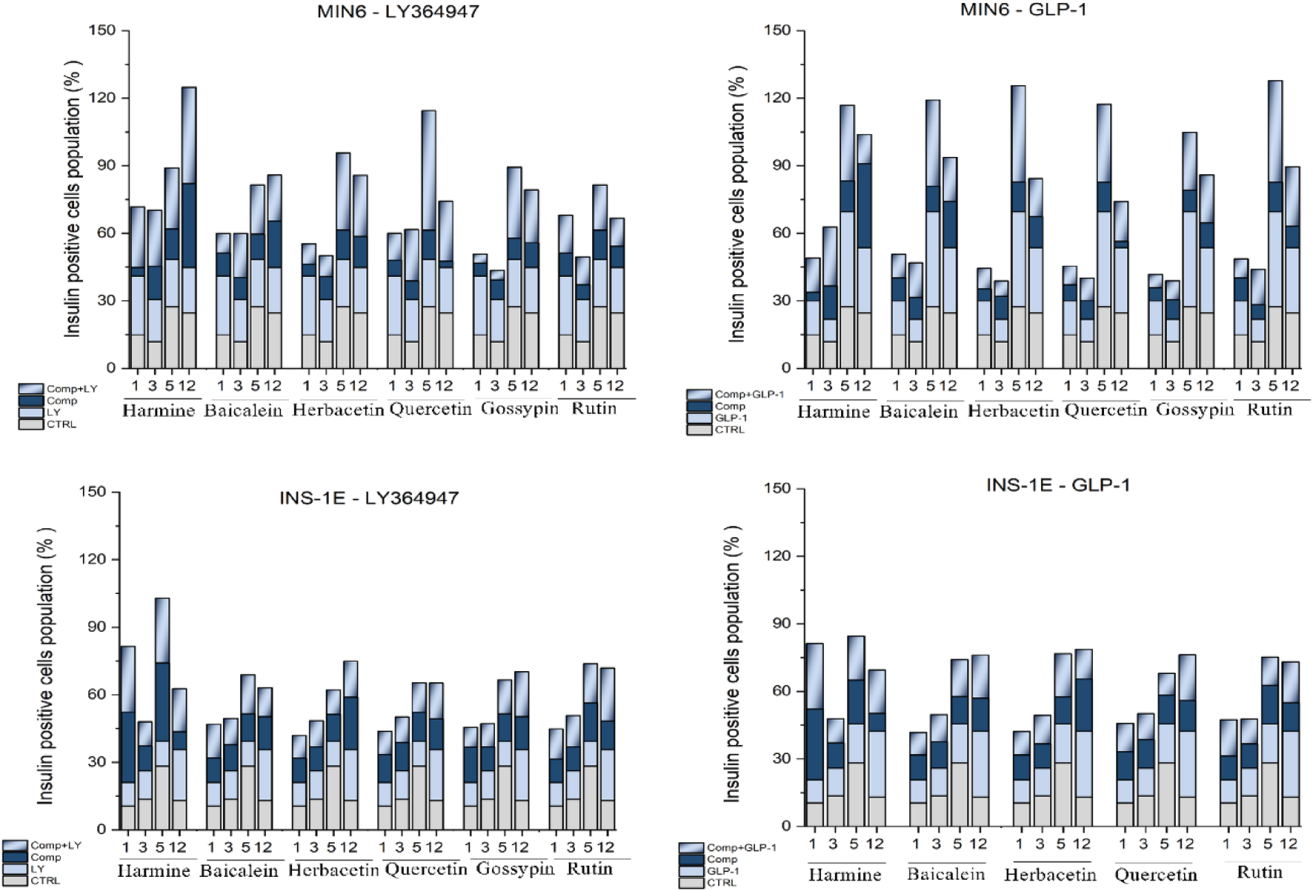
The impact of natural compounds, TGF-β inhibitor and GLP-1R agonist on MIN6 and INS1-E insulin expression level. The cells were incubated for 24 h with the most effective concentration of the compounds ((LY364947 (2.5 μM), GLP-1 (5 nM), harmine (1 μM), baicalein (1 μM), herbacetin (2.5 μM), quercetin (2.5 μM), gossypin (2.5 μM) and rutin (1 μM)). The medium was changed every 2 days. At selected days (1, 2, 3, 12) the cells were stained with the relevant antibodies and analysed by flow cytometry.

To determine the effects of the natural compounds, a TGF-β inhibitor and a GLP-1R agonist, on insulinoma cells function, we first investigated the effects on insulin production levels (Fig. 7). The experiment was carried out for 12 days to evaluate both the short- and long-term effect. Both cell lines (MIN6 and INS-1E) displayed a similar basal level of insulin which showed fluctuations over the course of the experiment and reached its highest level on day 5. MIN6 cells were more sensitive to treatment as manifested by increased insulin levels compared to treated INS-1E cells. In both cell lines harmine, a positive control, induced an increase in insulin level with the maximum effect noted at day 12 (37% vs. 24% in control) for MIN6, and day 1 (37% vs. 10%) or day 5 (34% vs. 28%) for INS-1E. The analysed natural compounds had a moderate effect but showed stabilization of insulin level over time. Only quercetin showed a noticeable decrease in insulin level on day 12. The incubation of MIN6 and INS-1E cells with GLP-1 led to a robust increase in insulin production level in the late stage of the experiment (day 5–12), but this effect was attenuated when harmine or natural compounds were administered simultaneously with GLP-1. The TGF-β inhibitor (LY364947) showed a less prominent but constant effect over the 12 days of the experiment. The effect was enhanced by supplementation with natural compounds (harmine and flavones) on long-term basis. The most effective was combination of LY364947 and harmine (day 12), baicalein (day 5), herbacetin (day 5 and 12), quercetin (day 5) or gossypin (day 5) clearly visible for MIN6 cells.

**Figure 8:**
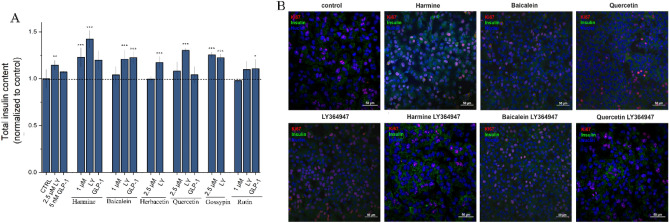
Natural compounds support insulin secretion modulated by TGF-β inhibitor and GLP-1R agonist in MIN6. (**A**) ELISA assay quantitative results of MIN6 insulin secretion. The cells were incubated for 24 h with natural compounds (harmine (1 μM), baicalein (1 μM), herbacetin (2.5 μM), quercetin (2.5 μM), gossypin (2.5 μM) and rutin (1 μM)) and/or LY364947 (2.5 μM), GLP-1 (5 nM). 48 h later the insulin secretion was stimulated by GSIS and ELISA assay was preformed. Data normalized to the control are presented as the arithmetic mean ± SEM. The asterisks denote p-values < *0.05, **0.01, ***0.001 compared to control. (**B**) Representative confocal images of MIN6 cells after treatment with selected compounds. After treatment with selected compounds and GSIS stimulation cells were stained with the appropriate antibodies against Ki-67 (red) and insulin (green). The nuclei were stained with Hoechst 33258 (blue). Scale bars, 50 μm.

This from prior paragraph observation prompted us to further study natural compounds in the context of MIN6 cells functionality. To get comprehensive results the MIN6 cells ability to secret insulin in response to glucose stimulation (glucose-stimulated insulin secretion, GSIS) was investigated by ELISA (Fig. 8A). The results indicated that LY364947 treatment led to an increase in insulin secretion compared to untreated cells. Both harmine and gossypin caused robust insulin secretion in response to glucose stimulation. A less pronounced effect was observed for cells after GLP-1 or quercetine treatment. No effect was observed for baicalein, herbacetin and rutin. A synergistic effect was observed for combined treatment of LY364947 and harmine, baicalein or quercetin. The observation was further confirmed by fluorescence confocal microscopy (Fig. 8B). For GLP-1 only combination with baicalein improve insulin secretion.

#### Natural compound activity in the advanced pancreatic islets models—hiPSC-derived beta cells organoids

**Figure 9:**
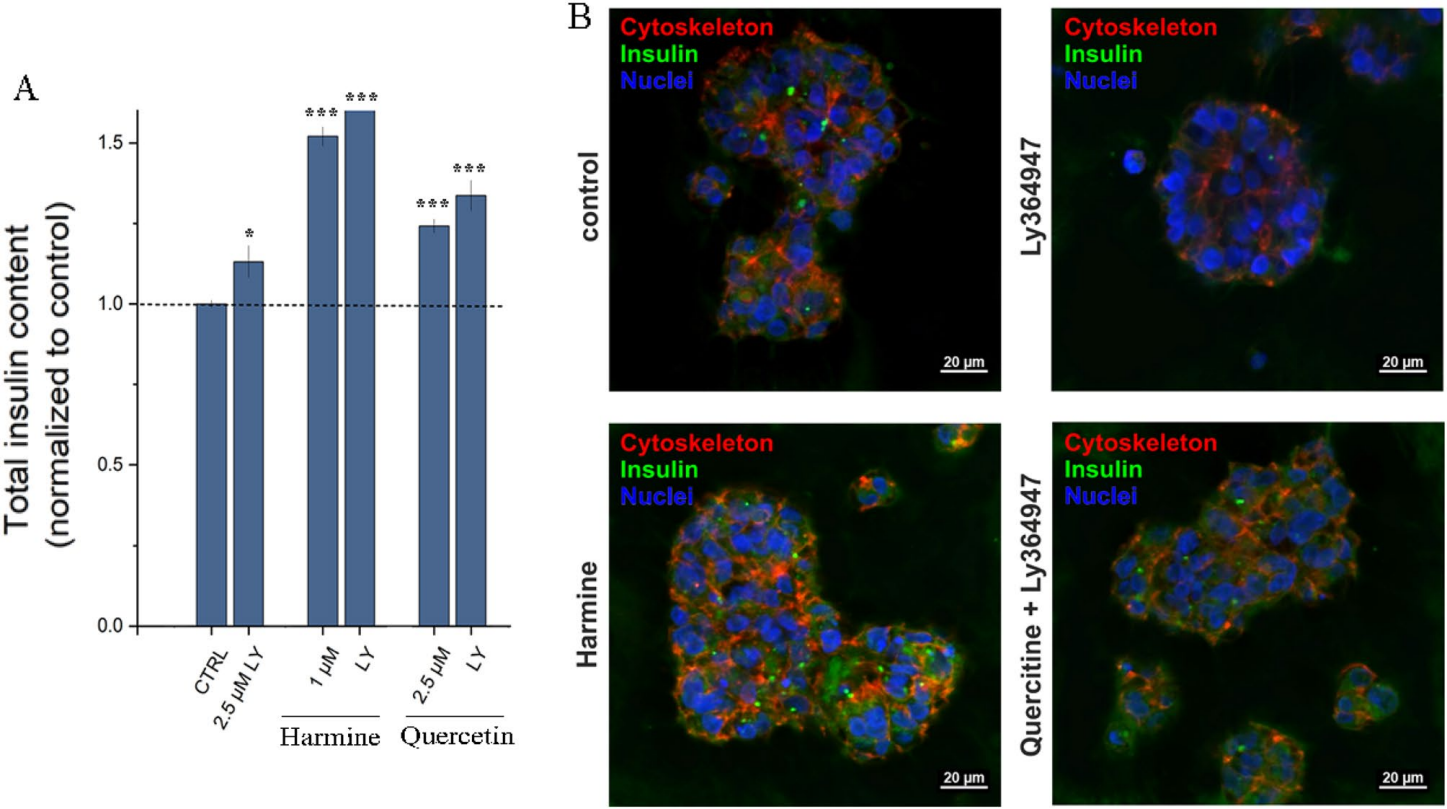
Quercetin and TGFβ inhibitor—LY364947 collectively support insulin release from hiPSC-derived pancreatic islet organoids. (**A**) The analysis of insulin secretion by ELISA assay. The hiPSC-derived beta cells organoids were incubated with the compound alone or in combination (harmine, quercetine or LY364947) or left untreated for 24 h. 48 h later the insulin secretion was stimulated by GSIS and ELISA assay was performed. Data normalized to the control are presented as the arithmetic mean ± SEM. The asterisks denote p-values <  *0.05, **0.01, ***0.001 compared to control. (**B**) Representative fluorescent confocal images of hiPSC-derived pancreatic islet organoids after incubation with harmine, quercetine and/or LY364947 and GSIS treatment. The organoids were immunostained for insulin (green) and cytoskeleton (red). The nuclei were stained with Hoechst 33258 (blue). Scale bars, 20 μm.

Reliable beta cells models are essential for the evaluation of small molecular compounds. The more advanced the model, the more valid the data, but it also implies longer analysis time, more work and expense. After the first round of analyses based on insulinoma cell lines, we proceeded to an advanced model of pancreatic islets—hiPSC-derived beta cells organoids. hiPSC-derived pancreatic islet organoids were produced in a 3D culture based on the developed protocol^37^. The process of differentiating hiPSCs into beta cells organoids took 21 days and yielded a limited number of organoids. Therefore, we selected only the most promising natural compound quercetin and TGF-β inhibitor (LY364947) for further study and compared it to harmine. First the impact of compounds treatment on organoids was analysed in the functional test of glucose-stimulated insulin secretion and quantified by ELISA assay (Fig. 9A). The highest insulin secretion level was observed for harmine, treatment with quercetin also led to a significant increase in insulin secretion by organoids. Both results were enhanced by LY364947 addition, while LY364947 alone gave a less pronounced effect. The obtained results were consistent with the confocal immunofluorescence analysis (Fig. 9B).

#### Chemical nature of flavones determines the biological potential

The kinase activity assay results revealed that both gossypin and quercetin are potent DYRK1A inhibitors. Based on cellular data, the biological potential was confirmed only for quercetin. To explain this discrepancy, we decided to check if the chemical nature of compounds could influence the obtained results. The potency and efficiency of drug candidates are strongly related to their lipophilicity. Thus, we determined the octanol/water partition coefficient (logP [octanol]/[water]), the index of compounds’ ability to differentially dissolve in a water and organic solvent mixture. The compound’s logP index for tested compounds ranged from -1.9 to 3.24 (Table 2) and reflects their chemical character. Both glycosides (rutin and gossypin) are characterized by the negative logP, owing to the hydrophilic character due to the presence of sugar moieties. The remaining tested compounds were characterized by positive logP, attributed to higher affinity to lipid/organic solvent and indicating the lipophilic character of compounds. LogP is a major element of the Lipinski’s Rule of 5, where ideally the values between 1.35 and 1.8 indicate good oral and intestinal absorption^88^. From tested compounds only quercetin and herbacetin fulfils the condition. The impact of logP values on the bioavailability of natural compounds may reduce their activity, which is likely reflected in our biological studies. LogP is an essential parameter that provides an indication of how a compound should be formulated to maintain its biological potency.

**Table 2:** LogP values for natural compounds determined by the shake-flask method.

Compound	LogP
Harmine	3.24
Baicalein	2.55
Gossypin	− 0.98
Herbacetin	1.72
Quercitin	1.40
Rutin	− 1.9

## Conclusions

The management of diabetes through controlled regeneration of beta cells remains attractive, yet not fully developed therapeutic strategy. Inhibition of DYRK1A is one of the best-documented approaches for controlled beta cells mass and function restoration. The most currently approved medications are derived from natural sources^89^. The reference DYRK1A inhibitor harmine is also a natural product.

Numerous studies have investigated the regulation of kinases by natural products, highlighting promising therapeutic prospects. Gossypin, for instance, inhibits NF-κB activation, a crucial cellular pathway in inflammation and cancer, by targeting IκBα kinase^90^. It also modulates LDLR expression through ERK kinase activation, indicating its potential as a hypocholesterolemic agent. Quercetin exhibits neuroprotective effects in Alzheimer's disease (AD) by targeting P13 kinase, AKT/PKB tyrosine kinase, and protein kinase C. It enhances insulin sensitivity and glucose metabolism, suggesting its potential in diabetes treatment. It increases glucose uptake by up-regulating estrogen receptor α, leading to enhanced phosphorylation of both phosphatidylinositol-3 kinase/Akt (PI3K/Akt) and AMP-activated protein kinase/Akt (AMPK/Akt) in skeletal muscle cells^91^. In AD, quercetin inhibits GSK3β activity, leading to reduced tau hyperphosphorylation, which addresses key AD markers^92–94^. Rutin is known for its anti-tumor and anti-inflammatory effects achieved by regulating p38 MAP kinase activity and limiting JNK phosphorylation, thus playing a protective role in UVB-induced skin damage^95,96^. The promising therapeutic potential of natural products in regulating kinase activity and their diverse mechanisms underscores their importance as well-established research subjects.

We investigated the use of flavones as potential starting points for development of antidiabetic, DYRK1A inhibitors. In the study we identified the structural and physicochemical requirements of flavones for a potent DYRK1A inhibition. The undoubted disadvantage of flavonoids is their complex landscape of multiple targets within cellular environments, leading to multiple off-target effects, potentially impacting cellular functions beyond the intended kinase inhibition. Therefore their chemical scaffold is only a starting point in hit-to-lead optimalization of DYRK1A inhibitors through a systematic and rational modification. Structure–activity relationship analysis has demonstrated the significant contribution of hydroxyl moieties of flavones in shaping their interaction with DYRK1A, contributing to the formation of unique network of hydrogen bonds. Interaction included both the classical DYRK1A-inhibitor anchor sites at residues from ATP-binding pocket—hinge region (Ser241, Leu242 and Glu239) and catalytic Lys188, and exclusive interaction with solvent-exposed region (Asn244 and Asp247) described previously only for few but potent and selective DYRK1A inhibitors^81,82^. Given the challenges in designing selective kinase inhibitors due to the significant sequence similarity in the kinases' ATP-binding pocket, exploring new interaction sites is of paramount importance. Thus Asn244 and Asp247 residues, that not only form the ATP’s ribose-binding pocket, but are additionally involved in the peptide substrate recognition^97^ provide the region with relevant variability to guide inhibitor selectivity^81^. The sugar moiety, present in gossypin, could support the direct DYRK1A-flavonoid interaction, but at the same time had a negative impact on bioavailability as observed in both our models, the insulinoma cells and pancreatic islets, as well as in previous study^98^. Contrary to glycosyloxyflavones (gossypin and rutin) the aglycones (quercetin, herbacetin and baicalein) not only support cell viability but also affect beta cells proliferation and support insulin production, thus restoring the functionality of insulinoma cells and beta cells organoids. In addition, the desired effect can be enhanced by combinatory treatment with TGF-β inhibitor. The above observation opens the opportunity of complex treatment of diabetes not limited to a single cellular pathway. Together, our data justifies that flavones, particularly quercetin, constitute promising starting points for development of antidiabetic DYRK1A inhibitors.

Flavones suffer from several liabilities that limit their effectiveness in vivo. The presence of a large number of polar hydroxyl moieties could significantly impact the bioavailability of a compound by hindering its passage through cell membranes, limiting distribution into lipophilic tissues, and potentially leading to rapid metabolism and shorter half-lives. Consequently, the presence of numerous hydroxyl moieties is concern and represents key points requiring modification. Examples of rational modification of synthetic hydroxyl derivatives of hit DYRK1A compounds (EGCG^99^ and DANDY)^100^ to fluorinated derivatives demonstrate that substitution not necessarily results in decreased inhibitory activity against DYRK1A, while significantly improved bioavailability and selectivity. In addition, DYRK1A is ubiquitous and plays an important role in the control of many signaling pathways. Thus, special attention should be paid to tissue-specific targeting of DYRK1A by selecting an relevant delivery of a potent inhibitor. The selectivity of the compound can be further improved by chemical modification of the scaffold, as it was shown previously for natural products: staurosporine^101^ and harmine^102^.

Based on our structural data and cellular validation, we foresee relatively straightforward path towards the therapeutic use of flavonoid-derived drugs against diabetes. Improvement of bioavailability and metabolic stability by chemical modification, together with tissue-specific formulations, can turn flavones from ubiquitous, non-selective inhibitors into leading molecules for future curative diabetes therapy.

### Supplementary Information

Supplementary Information.

## Data Availability

Crystal structures described in this article are available in the Protein Data Bank (PDB; https://www.rcsb.org/) with IDs, 8C3R and 8C3Q.
