# Supplementary material for: Binding mechanism and biological effects of flavone DYRK1A inhibitors for the design of new antidiabetics
Source: Sci Rep. 2023 Oct 23;13:18114. doi: 10.1038/s41598-023-44810-3 (PMC10593742; doi:10.1038/s41598-023-44810-3)
Supplement: Supplementary file 1 — Supplementary Information. [file 41598_2023_44810_MOESM1_ESM.pdf]

Supplementary Information

Binding mechanism and biological effects of flavone DYRK1A inhibitors for the design of new antidiabetics.

Katarzyna Pustelny[1], Przemyslaw Grygier[2], Agata Barzowska[2], Barbara Pucelik[1], Alex Matsuda[2], Krzysztof Mrowiec[2], Emilia Slugocka[3], Grzegorz M. Popowicz[4], Grzegorz Dubin[1], Anna Czarna[1,*]

[1]Malopolska Centre of Biotechnology, Jagiellonian University, Krakow, Poland

[2]Doctoral School of Exact and Natural Sciences, Jagiellonian University, Krakow, Poland

[3]Doctoral School of Medical and Health Sciences, Jagiellonian University Medical College, Krakow, Poland

[4]Institute of Structural Biology, Helmholtz Zentrum Munchen, Neuherberg, Germany

*** Correspondence:**

Anna Czarna

anna1.czarna@uj.edu.pl

Gronostajowa 7A, 30-387 Krakow, Poland

Phone: +48 12 664 5369

Fax: +48 12 664 6902

Table of Contents

Supplementary Table S1. Data collection and refinement statistics.

Supplementary Fig. S1. Electron density map, 2Fo-fc: +1.0σ (gray); Fo-Fc: +3.0σ (green); FoFc: -3.0σ (red). (A, B) Gossypin in DYRK1A/gossypin crystal structure (PDB ID 8C3R) for molecules A and B found in asymmetric unit, respectively, (C, D) rutin in DYRK1A/rutin crystal structure (PDB ID 8C3Q) for molecules A and B found in asymmetric unit, respectively. Figure was prepared in UCSF ChimeraX 1.6.1 (https://www.cgl.ucsf.edu/chimerax/) and GIMP 2.10.34 (https://www.gimp.org/)

Supplementary Fig. S2. Overall fold of DYRK1A (gray) in cartoon representation with rutin (PDB: 8C3Q) (magenta sticks) and gossypin (PDB: 8C3R) (cyan sticks) at the ATP-binding

pocket. Figure was prepared in UCSF ChimeraX 1.6.1 (https://www.cgl.ucsf.edu/chimerax/) and GIMP 2.10.34 (https://www.gimp.org/)

Supplementary Fig. S3. The contribution of water molecules to the binding of gossypin (A) ((PDB: 8C3R) (cyan sticks)) and rutin (B) ((PDB: 8C3Q) (magenta sticks)) in the active site of DYRK1A. Water molecules are represented as red spheres and hydrogen bonds are shown in gray dashes. Figure was prepared in UCSF ChimeraX 1.6.1 (https://www.cgl.ucsf.edu/chimerax/) and GIMP 2.10.34 (https://www.gimp.org/)

Supplementary Fig. S4. Uncropped Western-blots to Figure 1C

Supplementary Fig. S5. The control reaction for Cook activity assay.
The ability of selected compounds to inhibit DYRK1A was determined in the Cook activity assay, were ADP production was linked to puryvate kinas (PK) and lactate dehydrogenase (LDH) dependent NADH oxidation. In order to determine impact of analyzed compounds on PK and LDH enzymes the control reaction were carried out. The 85 µl of assay mixture (100 mM MOPS pH 6.8, 100 mM KCl, 10 mM $MgCl_2$, 1 mM phosphoenolpyruvate, 1 mM peptide substrate DYRKtide (RRRFRPASPLRGPPK, Caslo ApS), 1 mM β-Mercaptoethanol, 15 U/ml lactate dehydrogenase with 10 U/ml pyruvate kinase and 10.7 mM NADH) was mixed with 5 µl of a compound in DMSO (200 µM) or DMSO (control) and incubated for 10 minutes at room temperature. Then the reaction was started by simultaneous addition of 10 µl ATP (1280 µM) or ADP (1280 µM). In control reaction DYRK1A kinase was not included. The enzyme (PK and LDH) velocity was measured at 340 nm for 300 s at room temperature. The PK and LDH activity after incubation with DMSO (control) and addition of ADP was taken as a reference (100%) for the calculation of the residual activity after incubation with compounds.

Supplementary Fig. S6. Simulation Analysis Diagrams for: (I) rutin, (II) gossypin, (III) baicalein, (IV) herbacetin, (V) quercetin. (A) RMSD analysis of MD simulation trajectory for protein (blue), ligand-protein (red) and ligand (magenta). (B) RMSF plot for protein (blue) with the protein residues interacting with the ligand marked (green vertical bars). (C) RMSF plot for ligand (magenta) and 2D structure of the ligand colored by element (carbon-black, oxygen-red) indexed accordingly to the chart. Only heavy atoms are shown.

Supplementary Fig. S7. Atom types and particle charges for ligands after minimization with the OPLS4 force field, translated into atom symbols according to the Maestro User's Guide (2022-3 edition). The molecular representations with atom indexing are provided above the data.

Supplementary Table S1.

	DYRK1A/gossypin	DYRK1A/rutin
**PDB**	8C3R	8C3Q
**Wavelength**	1.0000	1.0332
**Resolution range**	48.36 - 2.06 (2.134 - 2.06)	49 - 2.32 (2.403 - 2.32)
**Space group**	P 63	P 63

**Unit cell**	132.126 132.126 90.485 (Å)<br>90 90 120 (°)	134.187 134.187 91.15 (Å)<br>90 90 120 (°)
**Total reflections**	719290 (38380)	296199 (29984)
**Unique reflections**	55250 (4133)	40460 (3951)
**Multiplicity**	13.0 (9.3)	7.3 (7.6)
**Completeness (%)**	99.39 (96.27)	99.88 (99.95)
**Mean I/sigma(I)**	18.6 (1.5)	12.4 (1.5)
**Wilson B-factor**	37.84	48.04
**R-merge**	0.091 (1.431)	0.099 (1.538)
**R-meas**	0.098 (1.603)	0.115 (1.777)
**CC1/2**	0.999 (0.518)	0.998 (0.487)
**Reflections used in refinement**	55222 (5322)	40432 (4017)
**Reflections used for R-free**	2763 (250)	2006 (210)
**R-work**	0.1855	0.1763
**R-free**	0.2091	0.2096
**Overall number of atoms**	6039	5746
**In macromolecules**	5535	5470
**In ligands**	84	86
**In waters**	420	190
**Protein residues**	691	688
**RMSD (bonds)**	0.004	0.007
**RMSD (angles)**	0.70	0.89
**Ramachandran favored (%)**	95.74	95.39
**Ramachandran allowed (%)**	4.26	4.61
**Ramachandran outliers (%)**	0.00	0.00
**Rotamer outliers (%)**	0.70	1.43
**Clashscore**	2.45	3.04
**Average B-factor**	50.00	60.47
**For macromolecules**	50.08	60.85
**For ligands**	45.36	47.62
**For solvent**	49.94	55.35

*Data for the highest resolution shell are shown in parentheses.

Supplementary Fig. S1.

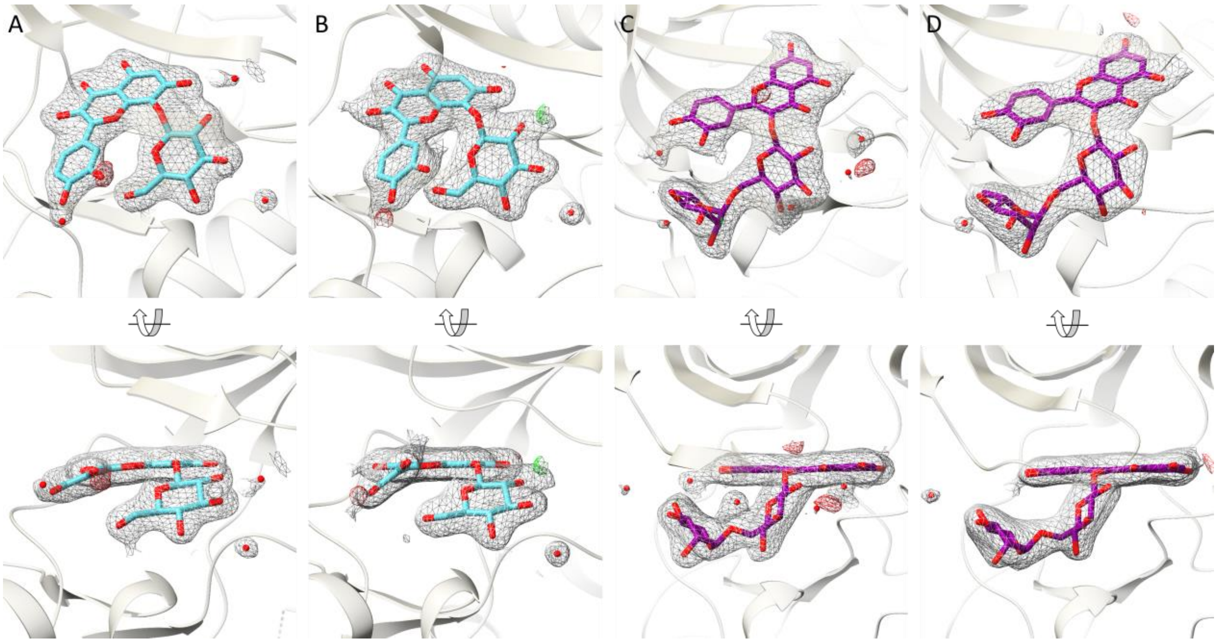

Supplementary Fig. S2.

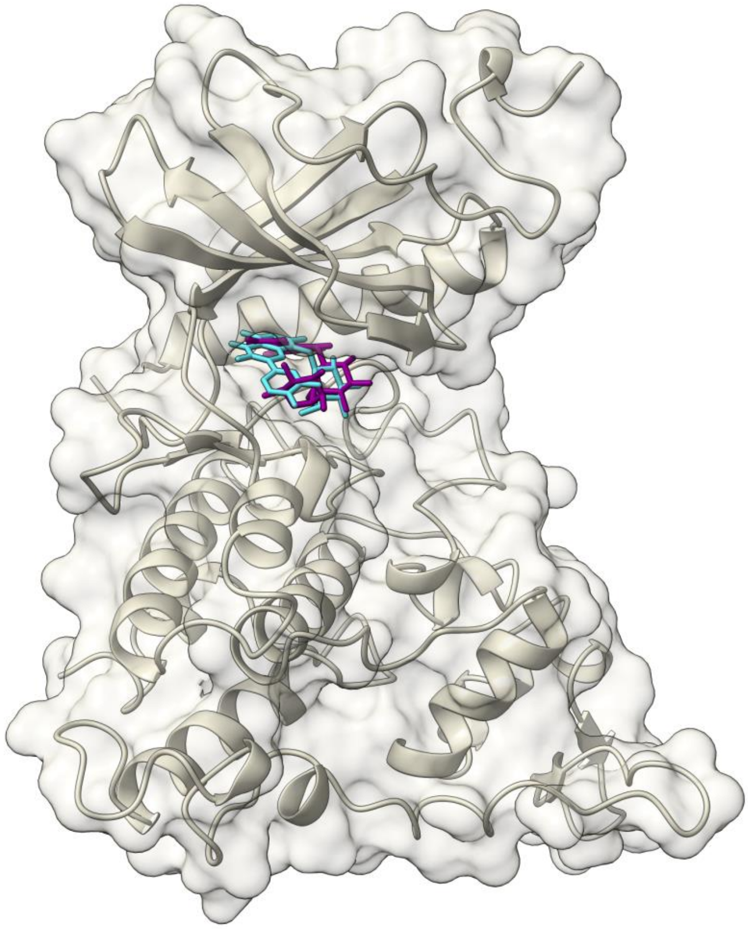

Supplementary Fig. S3.

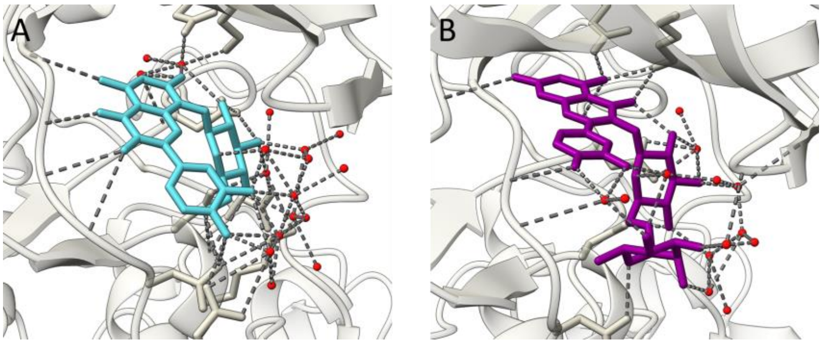

Supplementary Fig. S4.

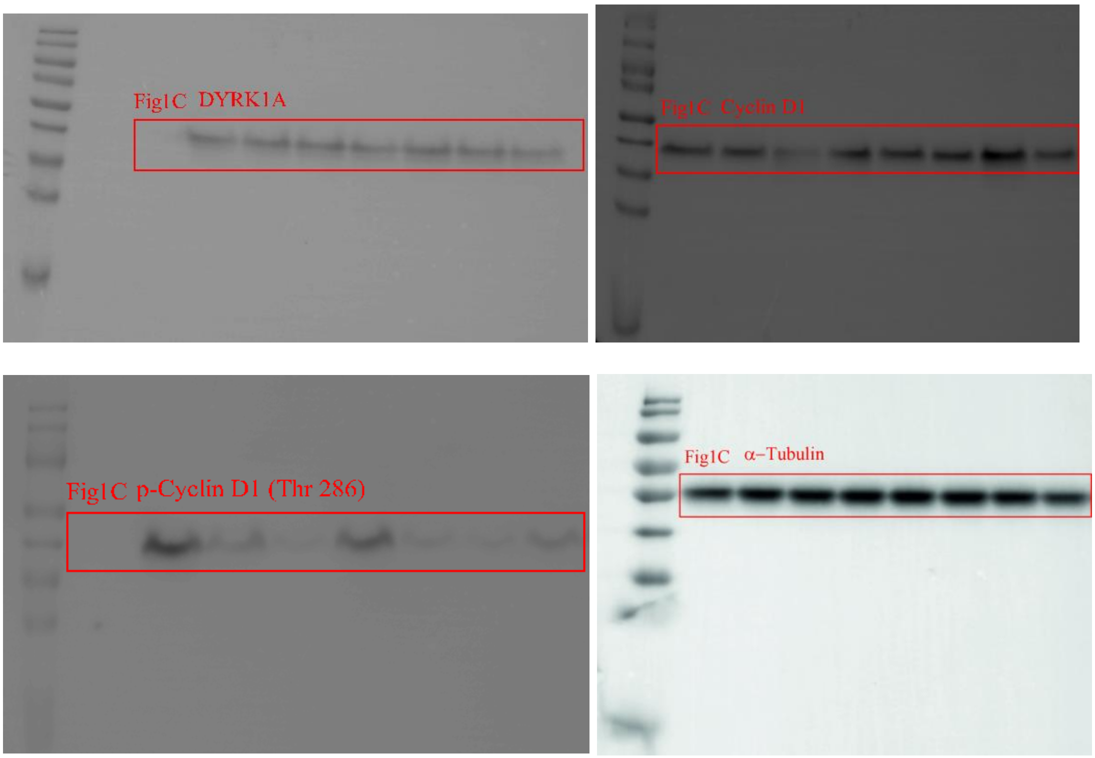

Supplementary Fig. S5.

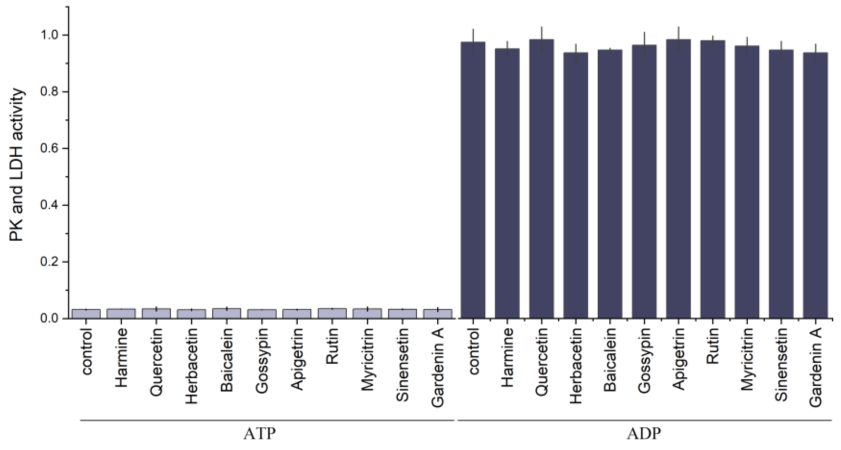

Supplementary Fig. S6.

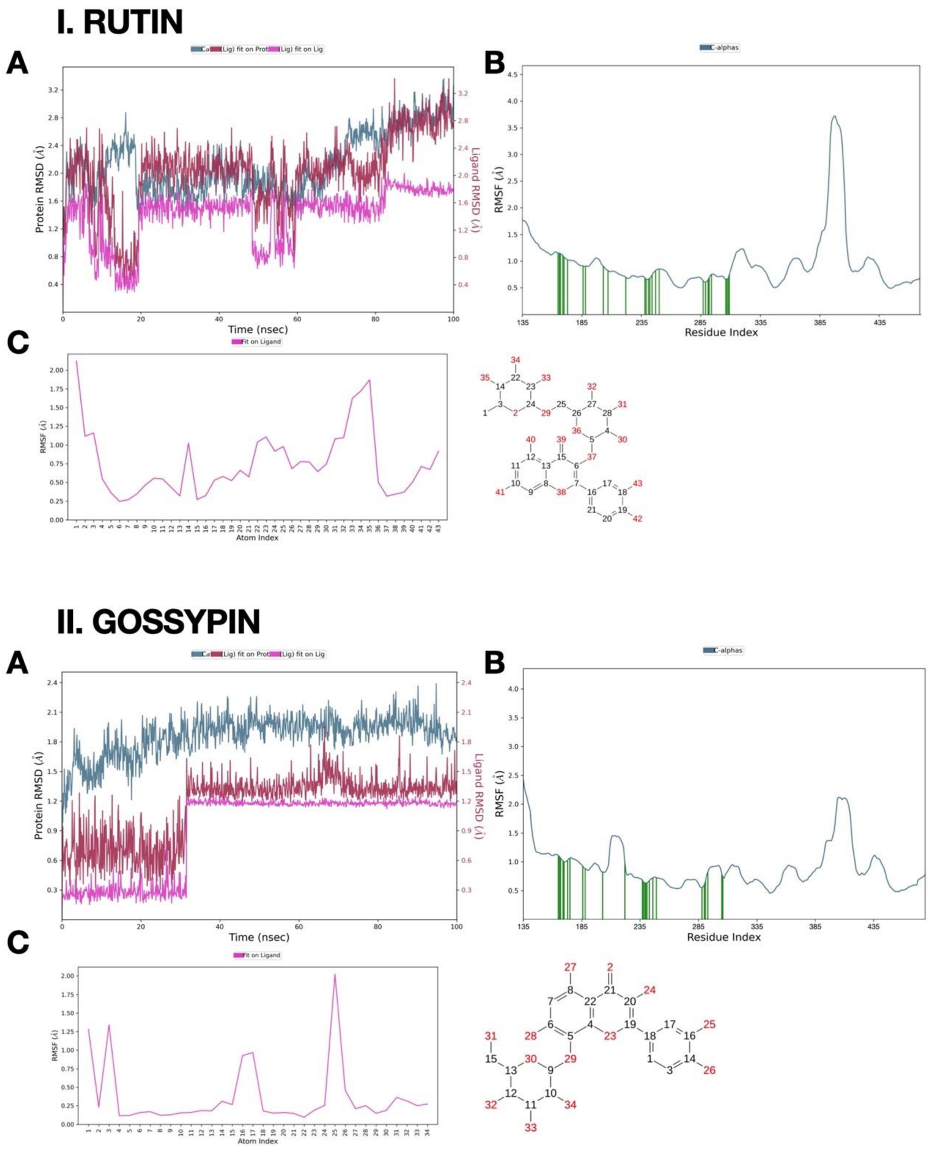

III. BAICALEIN

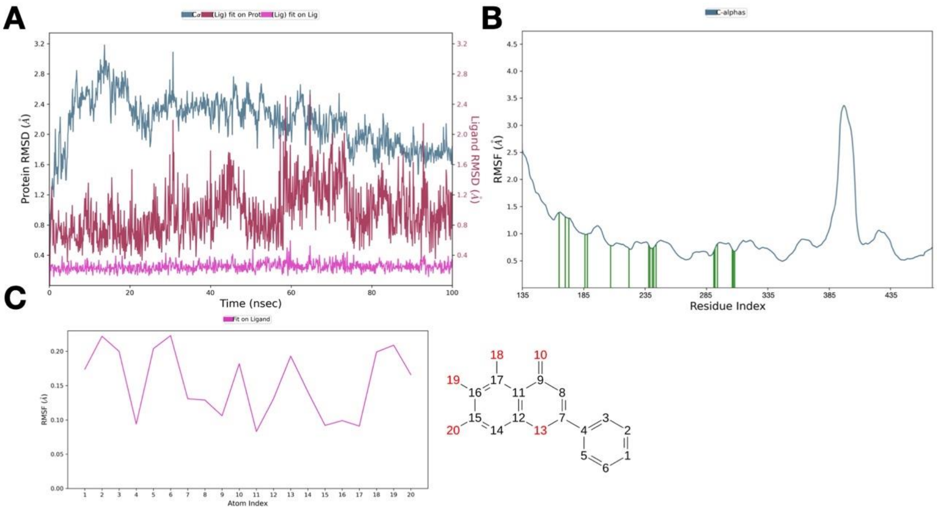

IV. HERBACETIN

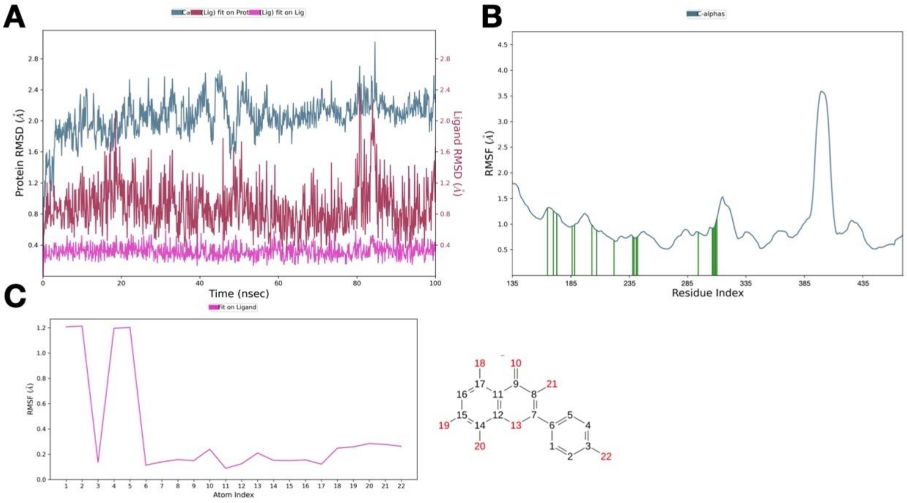

V. QUERCETIN

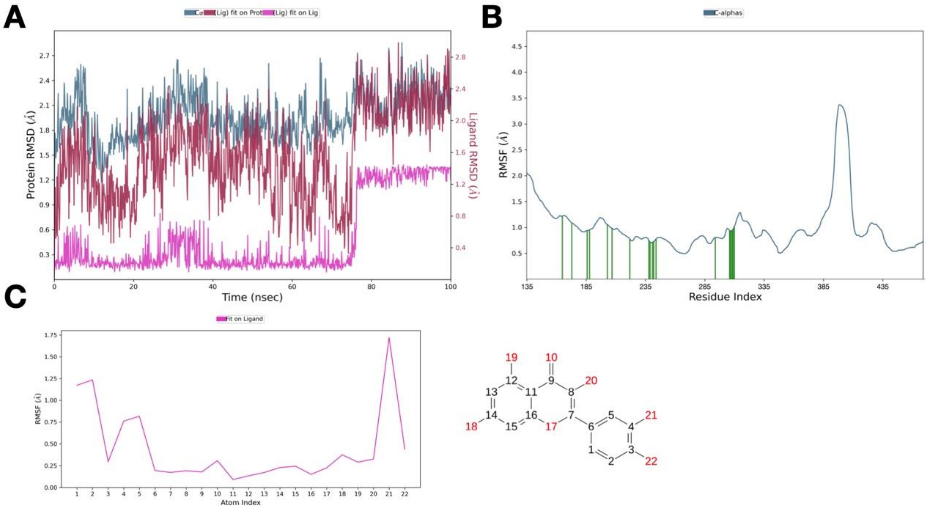

Supplementary Fig. S7.

I. RUTIN

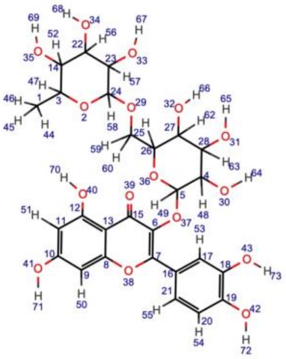

atom index	atom type	atom symbol	atom description	partial charge	atom index	atom type	atom symbol	atom description	partial charge
**1**	3	C3	carbon - sp3	-0.21217	**38**	16	O3	oxygen - single bond	-0.37612
**2**	16	O3	oxygen - single bond	-0.35779	**39**	15	O2	oxygen - double bond	-0.55762
**3**	3	C3	carbon - sp3	0.18474	**40**	16	O3	oxygen - single bond	-0.51783
**4**	3	C3	carbon - sp3	0.16536	**41**	16	O3	oxygen - single bond	-0.58240
**5**	3	C3	carbon - sp3	0.05729	**42**	16	O3	oxygen - single bond	-0.53368
**6**	2	C2	carbon - sp2	-0.33053	**43**	16	O3	oxygen - single bond	-0.53431
**7**	2	C2	carbon - sp2	0.24417	**44**	41	H1	H-electroneut	0.07322
**8**	2	C2	carbon - sp2	0.66671	**45**	41	H1	H-electroneut	0.07322
**9**	2	C2	carbon - sp2	-0.50544	**46**	41	H1	H-electroneut	0.07322
**10**	2	C2	carbon - sp2	0.37736	**47**	41	H1	H-electroneut	0.04886
**11**	2	C2	carbon - sp2	-0.44964	**48**	41	H1	H-electroneut	0.08710
**12**	2	C2	carbon - sp2	0.33900	**49**	41	H1	H-electroneut	0.15759
**13**	2	C2	carbon - sp2	-0.43023	**50**	41	H1	H-electroneut	0.22200
**14**	3	C3	carbon - sp3	0.13615	**51**	41	H1	H-electroneut	0.16077
**15**	2	C2	carbon - sp2	0.83562	**52**	41	H1	H-electroneut	0.08768
**16**	2	C2	carbon - sp2	-0.00186	**53**	41	H1	H-electroneut	0.14744
**17**	2	C2	carbon - sp2	-0.14723	**54**	41	H1	H-electroneut	0.14223
**18**	2	C2	carbon - sp2	0.18013	**55**	41	H1	H-electroneut	0.14752
**19**	2	C2	carbon - sp2	0.20193	**56**	41	H1	H-electroneut	0.07488
**20**	2	C2	carbon - sp2	-0.20514	**57**	41	H1	H-electroneut	0.07316
**21**	2	C2	carbon - sp2	-0.09026	**58**	41	H1	H-electroneut	0.18248
**22**	3	C3	carbon - sp3	0.14646	**59**	41	H1	H-electroneut	0.05847
**23**	3	C3	carbon - sp3	0.17830	**60**	41	H1	H-electroneut	0.05847
**24**	3	C3	carbon - sp3	0.03336	**61**	41	H1	H-electroneut	0.06260
**25**	3	C3	carbon - sp3	0.15430	**62**	41	H1	H-electroneut	0.07486
**26**	3	C3	carbon - sp3	0.16228	**63**	41	H1	H-electroneut	0.06984
**27**	3	C3	carbon - sp3	0.13931	**64**	42	H2	H-O (neutral)	0.42365
**28**	3	C3	carbon - sp3	0.14963	**65**	42	H2	H-O (neutral)	0.42138

**29**	16	O3	oxygen - single bond	-0.39633	**66**	42	H2	H-O (neutral)	0.42451
**30**	16	O3	oxygen - single bond	-0.64550	**67**	42	H2	H-O (neutral)	0.42544
**31**	16	O3	oxygen - single bond	-0.64196	**68**	42	H2	H-O (neutral)	0.42403
**32**	16	O3	oxygen - single bond	-0.64489	**69**	42	H2	H-O (neutral)	0.42926
**33**	16	O3	oxygen - single bond	-0.63001	**70**	42	H2	H-O (neutral)	0.40710
**34**	16	O3	oxygen - single bond	-0.64583	**71**	42	H2	H-O (neutral)	0.45284
**35**	16	O3	oxygen - single bond	-0.65620	**72**	42	H2	H-O (neutral)	0.40357
**36**	16	O3	oxygen - single bond	-0.34742	**73**	42	H2	H-O (neutral)	0.40501
**37**	16	O3	oxygen - single bond	-0.20410					

II. GOSSYPIN

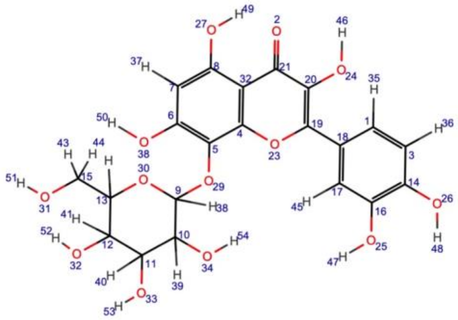

atom index	atom type	atom symbol	atom description	partial charge	atom index	atom type	atom symbol	atom description	partial charge
**1**	2	C2	carbon - sp2	-0.09120	**28**	16	O3	oxygen - single bond	-0.52518
**2**	15	O2	oxygen - double bond	-0.55662	**29**	16	O3	oxygen - single bond	-0.24473
**3**	2	C2	carbon - sp2	-0.20040	**30**	16	O3	oxygen - single bond	-0.36820
**4**	2	C2	carbon - sp2	0.66959	**31**	16	O3	oxygen - single bond	-0.65216
**5**	2	C2	carbon - sp2	-0.17885	**32**	16	O3	oxygen - single bond	-0.63605
**6**	2	C2	carbon - sp2	0.35257	**33**	16	O3	oxygen - single bond	-0.64983
**7**	2	C2	carbon - sp2	-0.44593	**34**	16	O3	oxygen - single bond	-0.64447
**8**	2	C2	carbon - sp2	0.33418	**35**	41	H1	H-electroneut	0.14815
**9**	3	C3	carbon - sp3	0.05111	**36**	41	H1	H-electroneut	0.14384
**10**	3	C3	carbon - sp3	0.16005	**37**	41	H1	H-electroneut	0.16448
**11**	3	C3	carbon - sp3	0.13510	**38**	41	H1	H-electroneut	0.14901
**12**	3	C3	carbon - sp3	0.13702	**39**	41	H1	H-electroneut	0.09261
**13**	3	C3	carbon - sp3	0.14376	**40**	41	H1	H-electroneut	0.07827
**14**	2	C2	carbon - sp2	0.20669	**41**	41	H1	H-electroneut	0.07528
**15**	3	C3	carbon - sp3	0.13978	**42**	41	H1	H-electroneut	0.06465
**16**	2	C2	carbon - sp2	0.18040	**43**	41	H1	H-electroneut	0.05934
**17**	2	C2	carbon - sp2	-0.15621	**44**	41	H1	H-electroneut	0.05934
**18**	2	C2	carbon - sp2	-0.01621	**45**	41	H1	H-electroneut	0.14728
**19**	2	C2	carbon - sp2	0.14110	**46**	42	H2	H-O (neutral)	0.41124

**20**	2	C2	carbon - sp2	-0.23885	**47**	42	H2	H-O (neutral)	0.40503
**21**	2	C2	carbon - sp2	0.86323	**48**	42	H2	H-O (neutral)	0.40502
**22**	2	C2	carbon - sp2	-0.42371	**49**	42	H2	H-O (neutral)	0.40803
**23**	16	O3	oxygen - single bond	-0.35521	**50**	42	H2	H-O (neutral)	0.40951
**24**	16	O3	oxygen - single bond	-0.49363	**51**	42	H2	H-O (neutral)	0.42549
**25**	16	O3	oxygen - single bond	-0.53279	**52**	42	H2	H-O (neutral)	0.42932
**26**	16	O3	oxygen - single bond	-0.53178	**53**	42	H2	H-O (neutral)	0.43210
**27**	16	O3	oxygen - single bond	-0.51626	**54**	42	H2	H-O (neutral)	0.43570

III. BAICALEIN

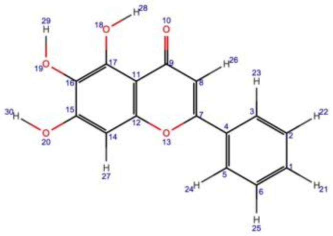

atom index	atom type	atom symbol	atom description	partial charge	atom index	atom type	atom symbol	atom description	partial charge
**1**	2	C2	carbon - sp2	-0.11500	**16**	2	C2	carbon - sp2	0.15000
**2**	2	C2	carbon - sp2	-0.11500	**17**	2	C2	carbon - sp2	0.15000
**3**	2	C2	carbon - sp2	-0.11500	**18**	16	O3	oxygen - single bond	-0.58500
**4**	2	C2	carbon - sp2	0.00000	**19**	16	O3	oxygen - single bond	-0.58500
**5**	2	C2	carbon - sp2	-0.11500	**20**	16	O3	oxygen - single bond	-0.58500
**6**	2	C2	carbon - sp2	-0.11500	**21**	41	H1	H- electroneut	0.11500
**7**	2	C2	carbon - sp2	0.08500	**22**	41	H1	H- electroneut	0.11500
**8**	2	C2	carbon - sp2	-0.11500	**23**	41	H1	H- electroneut	0.11500
**9**	2	C2	carbon - sp2	0.58500	**24**	41	H1	H- electroneut	0.11500
**10**	15	O2	oxygen - double bond	-0.47000	**25**	41	H1	H- electroneut	0.11500
**11**	2	C2	carbon - sp2	-0.11500	**26**	41	H1	H- electroneut	0.11500
**12**	2	C2	carbon - sp2	0.08500	**27**	41	H1	H- electroneut	0.11500
**13**	16	O3	oxygen - single bond	-0.17000	**28**	42	H2	H-O (neutral)	0.43500
**14**	2	C2	carbon - sp2	-0.11500	**29**	42	H2	H-O (neutral)	0.43500
**15**	2	C2	carbon - sp2	0.15000	**30**	42	H2	H-O (neutral)	0.43500

IV. HERBACETIN

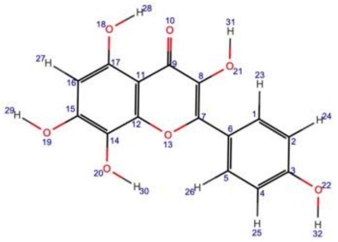

atom index	atom type	atom symbol	atom description	partial charge	atom index	atom type	atom symbol	atom description	partial charge
**1**	2	C2	carbon - sp2	-0.11500	**17**	2	C2	carbon - sp2	0.15000
**2**	2	C2	carbon - sp2	-0.11500	**18**	16	O3	oxygen - single bond	-0.58500
**3**	2	C2	carbon - sp2	0.15000	**19**	16	O3	oxygen - single bond	-0.58500
**4**	2	C2	carbon - sp2	-0.11500	**20**	16	O3	oxygen - single bond	-0.58500
**5**	2	C2	carbon - sp2	-0.11500	**21**	16	O3	oxygen - single bond	-0.40400
**6**	2	C2	carbon - sp2	0.00000	**22**	16	O3	oxygen - single bond	-0.58500
**7**	2	C2	carbon - sp2	0.08500	**23**	41	H1	H-electroneut	0.11500
**8**	2	C2	carbon - sp2	0.04880	**24**	41	H1	H-electroneut	0.11500
**9**	2	C2	carbon - sp2	0.58500	**25**	41	H1	H-electroneut	0.11500
**10**	15	O2	oxygen - double bond	-0.47000	**26**	41	H1	H-electroneut	0.11500
**11**	2	C2	carbon - sp2	-0.11500	**27**	41	H1	H-electroneut	0.11500
**12**	2	C2	carbon - sp2	0.08500	**28**	42	H2	H-O (neutral)	0.43500
**13**	16	O3	oxygen - single bond	-0.17000	**29**	42	H2	H-O (neutral)	0.43500
**14**	2	C2	carbon - sp2	0.15000	**30**	42	H2	H-O (neutral)	0.43500
**15**	2	C2	carbon - sp2	0.15000	**31**	42	H2	H-O (neutral)	0.35520
**16**	2	C2	carbon - sp2	-0.11500					

V. QUERCETIN

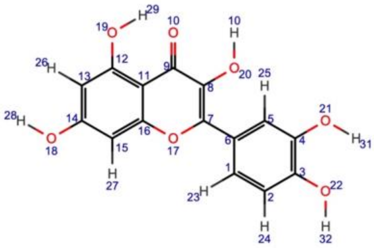

atom index	atom type	atom symbol	atom description	partial charge	atom index	atom type	atom symbol	atom description	partial charge
**1**	2	C2	carbon - sp2	-0.08762	**17**	16	O3	oxygen - single bond	-0.36863
**2**	2	C2	carbon - sp2	-0.18951	**18**	16	O3	oxygen - single bond	-0.58140
**3**	2	C2	carbon - sp2	0.13471	**19**	16	O3	oxygen - single bond	-0.51884
**4**	2	C2	carbon - sp2	0.18666	**20**	16	O3	oxygen - single bond	-0.51675
**5**	2	C2	carbon - sp2	-0.09034	**21**	16	O3	oxygen - single bond	-0.54721
**6**	2	C2	carbon - sp2	0.01160	**22**	16	O3	oxygen - single bond	-0.56606

**7**	2	C2	carbon - sp2	0.20834	**23**	41	H1	H-electroneut	0.14097
**8**	2	C2	carbon - sp2	-0.25101	**24**	41	H1	H-electroneut	0.13634
**9**	2	C2	carbon - sp2	0.82812	**25**	41	H1	H-electroneut	0.15792
**10**	15	O2	oxygen - double bond	-0.59933	**26**	41	H1	H-electroneut	0.16110
**11**	2	C2	carbon - sp2	-0.43572	**27**	41	H1	H-electroneut	0.22353
**12**	2	C2	carbon - sp2	0.34246	**28**	42	H2	H-O (neutral)	0.45301
**13**	2	C2	carbon - sp2	-0.45328	**29**	42	H2	H-O (neutral)	0.40812
**14**	2	C2	carbon - sp2	0.38183	**30**	42	H2	H-O (neutral)	0.43428
**15**	2	C2	carbon - sp2	-0.50831	**31**	42	H2	H-O (neutral)	0.41698
**16**	2	C2	carbon - sp2	0.67469	**32**	42	H2	H-O (neutral)	0.41334
